# Inflammatory biomarker response to GLP-1 receptor agonists versus other glucose-lowering medications in patients with type 2 diabetes: a systematic review and meta-analysis

**DOI:** 10.3389/fendo.2025.1734549

**Published:** 2026-01-15

**Authors:** Tariq Alrasheed, Mohamed E. A. Mostafa, Mohammed A Madkhali, Hesham A Khairy

**Affiliations:** 1 Department of Internal Medicine, Faculty of Medicine, University of Tabuk, Tabuk, Saudi Arabia; 2 Department of Anatomy, Faculty of Medicine, University of Tabuk, Tabuk, Saudi Arabia; 3 Department of Internal Medicine, Division of Endocrinology, Faculty of Medicine, Jazan University, Jazan, Saudi Arabia; 4 Department of Anatomy and Physiology, College of Medicine, Imam Mohammad Ibn Saud Islamic University (IMSIU), Riyadh, Saudi Arabia

**Keywords:** crp, GLP-1 RA, IL-6, inflammatory biomarker, MDA, T2D, TNF-α

## Abstract

**Background:**

Type 2 diabetes (T2D) is strongly linked to chronic inflammation and oxidative stress, which drive cardiovascular complications. Glucagon-like peptide-1 receptor agonists (GLP-1 RAs) demonstrate cardioprotective benefits that may extend beyond glycemic control, but their effects on key inflammatory and oxidative stress biomarkers compared to other glucose-lowering medications remain inconsistently reported across individual studies.

**Methods:**

A systematic review and meta-analysis of randomized controlled trials (RCTs) was conducted. Databases were searched for RCTs comparing GLP-1 RAs against other antidiabetic drugs or placebo in adults with T2D, reporting changes in inflammatory biomarkers (C-reactive protein [CRP], interleukin-6 [IL-6], tumor necrosis factor-alpha [TNF-α]) or the oxidative stress marker malondialdehyde (MDA). Data were pooled using a random-effects model, and outcomes were stratified by comparator type (placebo, insulin, other oral antidiabetic drugs [OADs]).

**Results:**

Forty RCTs (n=6029 participants) were included. GLP-1 RA therapy significantly reduced CRP levels compared to placebo (SMD = -0.59; 95% CI: -0.84 to -0.34) and other OADs (SMD = -1.06; 95% CI: -1.64 to -0.47). A significant reduction in TNF-α was observed versus placebo (SMD = -0.61; 95% CI: -0.89 to -0.32) and oral antidiabetic drugs add on (SMD = -1.62; 95% CI: -2.86 to -0.38). Data for MDA were limited and showed a non-significant trend toward reduction. GLP-1 RAs also significantly reduced IL-6 versus insulin (SMD = -0.24; 95% CI: -0.46 to -0.02). While significant heterogeneity was noted across the analyses, sensitivity analyses confirmed a consistent direction of effect, reinforcing the class-wide anti-inflammatory properties of GLP-1 RAs.

**Conclusion:**

GLP-1 RAs significantly improve key biomarkers of systemic inflammation (CRP, TNF-α) in patients with T2D compared to various active comparators and placebo. These pleiotropic effects provide a mechanistic rationale for their cardiovascular benefits and support their use as a multifaceted therapeutic strategy in T2D management.

**Systematic review registration:**

https://www.crd.york.ac.uk/PROSPERO/view/CRD420251157476, identifier CRD420251157476.

## 1 Introduction

Over the past few decades, the prevalence of type 2 diabetes (T2D) has increased alarmingly, leading to a global epidemic and a major public health concern due to its close correlation with an elevated risk of cardiovascular disease (CVD), the primary cause of morbidity and mortality in this population (1). A key pathophysiological element involves elevated oxidative stress and chronic, low-grade inflammation, which significantly contribute to the development of atherosclerosis, endothelial dysfunction, and microvascular damage (2–5).

This inflammatory and oxidative burden can be assessed through established biomarkers. High-sensitivity C-reactive protein (hs-CRP) is a well-known systemic inflammatory marker and a strong predictor of future cardiovascular events (6). Pro-inflammatory cytokines, such as interleukin-6 (IL-6) and tumor necrosis factor-alpha (TNF-α), are central to the inflammatory cascade, directly influencing insulin resistance, endothelial cell activation, and plaque instability (7, 8). Concurrently, oxidative stress, indicated by markers like malondialdehyde (MDA), results from an imbalance between reactive oxygen species and antioxidant defenses and is linked to diabetes-related complications (9, 10).

Conventional T2D treatments, including Sodium-Glucose Co-transporter-2 inhibitors (SGLT2 inhibitors), metformin, sulfonylureas, dipeptidyl peptidase 4 inhibitors (DPP-4 inhibitors), and insulin, primarily focus on glycemic control but exert varying and often modest effects on the underlying inflammatory state (11). For example, while metformin may have minor anti-inflammatory properties (12), it appears less effective than agents like exenatide in reducing hs-CRP (13). Similarly, insulin therapy, though essential, has rarely demonstrated superior efficacy in lowering inflammatory markers like TNF-α and CRP compared to other treatments and is associated with weight gain (14, 15). This underscores a significant therapeutic gap for interventions that address both hyperglycemia and the inflammatory pathways heightening cardiovascular risk.

Glucagon-like peptide-1 receptor agonists (GLP-1 RAs) represent a transformative class of treatment. By mimicking the incretin hormone GLP-1, medications like liraglutide, exenatide, and dulaglutide enhance glucose-dependent insulin secretion, suppress glucagon, and promote weight loss (1). Beyond these metabolic benefits, large-scale cardiovascular outcome trials (CVOTs) have consistently demonstrated that several GLP-1 RAs significantly reduce major adverse cardiovascular events, suggesting cardioprotective effects that extend beyond glycemic and weight control (16). A leading hypothesis posits that these advantages are mediated, at least in part, by direct anti-inflammatory and anti-oxidative properties.

Consequently, numerous randomized controlled trials (RCTs) have investigated the impact of GLP-1 RAs on relevant biomarkers. The findings, however, have been inconsistent. Some studies report significant reductions in TNF-α and CRP with GLP-1 RA therapy compared to placebo or other antidiabetic medications (6, 15, 17, 18), while others show non-significant or conflicting results (19, 20). For instance, while liraglutide significantly lowered TNF-α in one study (18), another found no significant change versus a DPP-4 inhibitor (20). Similarly, evidence for oxidative stress markers like MDA is promising but varied, with some trials showing clear superiority of GLP-1 RAs over insulin or metformin (9, 21, 22). This heterogeneity likely stems from differences in study populations, specific GLP-1 RAs, comparator treatments, and trial durations, highlighting the need for a comprehensive synthesis.

Given the contradictory data from individual trials, a systematic, quantitative synthesis is essential to clarify the true effect of GLP-1 RAs on inflammation and oxidative stress. A meta-analysis can overcome the limitations of single studies by providing a more precise and reliable estimate of treatment impact. Therefore, the primary aim of this systematic review and meta-analysis is to rigorously evaluate data from available RCTs to determine the effect of GLP-1 RA therapy on key inflammatory biomarkers (CRP, IL-6, TNF-α) and the oxidative stress marker (MDA) in patients with T2D. By stratifying the analysis by comparator type we seek to provide a clearer understanding of the relative efficacy of GLP-1 RAs in modulating these critical pathways and to elucidate the mechanisms underlying their established cardiovascular benefits.

## 2 Methodology

The research protocol for this study was registered with the International Prospective Register of Systematic Reviews (PROSPERO) with registration number CRD420251157476. The Preferred Reporting Items for Systematic Reviews and Meta Analyses (PRISMA) checklist criteria (23) were followed in order to ensure a systematic approach to the search procedure and reporting of the results shown in Supplementary Appendix S2.

### 2.1 Search strategy

From the inception to August 1, 2025, we searched four electronic databases for relevant literature: PubMed/MEDLINE, Embase, Cochrane CENTRAL, Web of Science. Only human randomized controlled trials (RCTs) published in English were included in the search. For two key concepts, comprehensive search strings were created using a combination of free-text keywords and controlled vocabulary (MeSH/Emtree terms): (1) GLP-1 receptor agonists (including specific drug names) and (2) inflammatory biomarkers (C-reactive protein, interleukin-6, and tumor necrosis factor-alpha). The full syntax for each database, including line-by-line strategies with resultant hits, is provided in Supplementary Material S3.

The World Health Organization International Clinical Trials Registry Platform (ICTRP) and the ClinicalTrials.gov registry were also reviewed to find unpublished or ongoing trials. The reference lists of included studies and relevant systematic reviews were manually searched to ensure thorough coverage.

### 2.2 Study selection criteria

#### 2.2.1 Participants/population

Adults (≥18 years old) diagnosed with T2D were included in the study. No restrictions were applied on the basis of race, ethnicity, or sex. Comorbid conditions including obesity, high blood pressure, or heart disease may be present in participants.

#### 2.2.2 Intervention(s)

Any GLP-1 receptor agonist that has been approved for the treatment of type 2 diabetes was used as the intervention of interest. It can be given at any dose, by any method, and for any period of time. Both short-acting and long-acting GLP-1 RAs were included.

#### 2.2.3 Comparator(s)/control

Other glucose-lowering drugs used to treat type 2 diabetes, such as insulin, sulfonylureas, DPP-4 inhibitors, SGLT2 inhibitors, metformin, and thiazolidinediones, will serve as comparators.

#### 2.2.4 Exclusion criteria

Studies involving patients with type 1 diabetes, gestational diabetes, or other specific types of diabetes were excluded (unless data for individuals with type 2 diabetes can be retrieved independently). Additionally excluded are studies conducted on individuals who are on systemic anti-inflammatory or immunosuppressive drugs, or who have active inflammatory conditions (such as rheumatoid arthritis or acute infections). We excluded case reports, case series, reviews, meta-analyses, editorials, conference papers, animal experiments, and *in vitro* research.

#### 2.2.5 Main outcomes

The main outcomes are changes in circulating inflammatory biomarkers, specifically high-sensitivity C-reactive protein (hs-CRP), Interleukin-6 (IL-6), and Tumor necrosis factor-alpha (TNF-α). Both absolute changes from baseline and percent changes will be considered. Data on other inflammatory markers (such as IL-1β, IL-8, adiponectin, etc.) will also be collected if available.

#### 2.2.6 Data extraction

Using a standardized data extraction form, two authors separately gathered relevant data from each included study. Discussions were utilized to resolve any disputes. Details of the interventions (specific GLP-1 RA and comparator drugs doses), participant characteristics (sample size, age, sex, baseline HbA1c, and comorbidities), study characteristics (first author, publication year, country, duration), and outcome data were included in the extracted data.

#### 2.2.7 Risk of bias (quality) assessment

Two authors independently assessed the risk of bias for each included RCT using the Cochrane Risk of Bias tool (RoB 2) (24). This tool evaluates several domains: randomization process, deviations from intended interventions, missing outcome data, measurement of the outcome, and selection of the reported result. Each domain was rated as “low risk,” “some concerns,” or “high risk,” leading to an overall trial risk of bias judgment. Disagreements were resolved through discussion or by a third author.

#### 2.2.8 Strategy for data synthesis

A meta-analysis was performed to quantitatively combine the results across studies for each outcome. A random-effects model was used to account for heterogeneity. Heterogeneity was assessed using the I² statistic and Cochran’s Q test and I² value greater than 50% was considered indicative of significant heterogeneity. For each outcome, a forest plot was performed to show individual study effects, and the pooled effect estimate with a 95% confidence interval. Separate meta-analyses were performed for each major comparator class. Sensitivity analyses were carried out by excluding studies with a high potential for bias and/or using leave-one-out analysis to evaluate the influence of individual studies.

## 3 Results

### 3.1 Study selection and characteristics

The initial systematic literature search across PubMed, Embase, and the Cochrane Library yielded 1,148 records. After removing 397 duplicates, 761 titles and abstracts were screened for eligibility. Of these, 377 records were excluded as they did not meet the inclusion criteria (e.g., wrong population, intervention, or study design). The full texts of the remaining 384 articles were assessed for eligibility. At the full-text screening stage, 344 articles were excluded for the following reasons: lack of relevant biomarker data (n=208), non-randomized study design (n=86), publication as conference abstracts without full data (n=35), and an ineligible study population (n=15). Finally, 40 RCTs met the full inclusion criteria and were included in this systematic review and meta-analysis. The detailed study selection process is illustrated in the PRISMA flow diagram (**Supplementary Figure S1**).

The 40 included studies enrolled a total of 6029 participants with type 2 diabetes (T2D). The characteristics of these studies are summarized in **Table 1**. The studies were published between 2010 and 2023. Geographic distribution was diverse, with a high number conducted in China (n=19), followed by Italy (n=4), Greece (n=3), and Japan (n=3). Study durations ranged from 2 weeks to 3 years, with 12 weeks and 24–26 weeks being the most common follow-up periods. The GLP-1 receptor agonists (GLP-1 RAs) investigated were predominantly liraglutide (23 studies) and exenatide (13 studies), with a smaller number examining dulaglutide (2 studies) and none examined semaglutide. Our inclusion criteria, which required protocol-specified measurement of at least one of the target inflammatory biomarkers (CRP, IL-6, TNF-α, or MDA) and publishing as a full-length RCT report, are reflected in the lack of semaglutide trials. These markers were not prospectively included in the major semaglutide CVOTs (SUSTAIN-6, PIONEER-6, SELECT). Instead of reporting distinct RCT results, the only data available are exploratory *post-hoc* analyses of hs-CRP that pool individual-level data across trials [Mosenzon et al., 2022 (25); Verma et al., 2023 (26)].

**Table 1:** Characteristics of the included studies.

Study (First author, Year)	Country	Sample size (n)	Participant characteristics	Intervention	Comparator	Biomarkers cnalyzed	Duration	Key outcomes summary
Ahmad, 2021 (16)	UK	61	Age: 43.8 ± 6.5; Sex: Lirag: 53.6% (15/28). Sita: 42.4% (14/33); HbA1c: 7.5%; Comorbidities: Obesity (BMI ≥30)	Liraglutide 1.8 mg	Sitagliptin 100 mg	VEGF, SDF-1α, CPCs, NO, CRP, IL-6, TNFα, AGEs	26 weeks	Liraglutide demonstrated a more favorable impact on vascular endothelial growth factor (VEGF) and stromal cell-derived factor-1 alpha (SDF-1α) compared to sitagliptin, suggesting differential cardiovascular effects. No significant differences were observed for other inflammatory markers.
Anholm et al., 2019 (6)	Denmark	41	Age: 62.3 ± 7.6; Sex: 79%; HbA1c: 6.4%; Comorbidities: Stable CAD, newly diagnosed T2D, BMI ≥ 25	Liraglutide + metformin	Placebo + metformin	CRP, TNF-α	12 + 12 weeks	In patients with stable CAD and new-onset T2D, the combination of liraglutide and metformin significantly reduced CRP levels but not TNF-α, alongside improvements in the atherogenic LDL lipid profile.
Bi, 2014 (27)	China	33	Age: 52.7 ± 1.7; Sex: Exe: 63.6% (7/11), Ins: 45.5% (5/11), Pio: 36.4% (4/11) M; HbA1c: 8.7%; Comorbidities: Drug-naive T2D	Exenatide	Insulin, Pioglitazone	IHF (Intrahepatic fat), VF (Visceral fat), SF (Subcutaneous fat)	6 months	All treatments significantly reduced intrahepatic fat. Exenatide uniquely reduced both visceral and subcutaneous fat, suggesting that early glycemic control with GLP-1 RAs can slow the progression of fatty liver disease.
Bouchi et al., 2017 (17)	Japan	19	Age: ~58.5 ± 19; Sex: 63%; HbA1c: ~8.2%; Comorbidities: T2D on insulin, BMI ≥ 25	Liraglutide + Insulin	Insulin alone	CRP, Hepatic fat, Albuminuria	24 weeks	Liraglutide add-on therapy significantly reduced CRP levels, visceral adiposity, and hepatic fat accumulation, indicating a role in mitigating micro-inflammation and ectopic fat deposition.
Bunck et al., 2010 (9)	Finland, Sweden, Netherl-ands	60	Age: ~58.4 ± 1.4; Sex: 63.9; HbA1c: ~7.6%; Comorbidities: T2D on metformin	Exenatide + metformin	Insulin Glargine + metformin	MDA, oxLDL	1 year	Exenatide treatment led to a significant reduction in postprandial excursions of oxidative stress markers (MDA, oxLDL) compared to insulin glargine, highlighting beneficial effects beyond glycemic control.
Derosa et al., 2010 (19)	Italy	128	Age: ~56.5 ± 7.5; Sex: 47.6; HbA1c: ~8.8%; Comorbidities: T2D uncontrolled on metformin	Exenatide + metformin	Glibenclamide + metformin	Hs-CRP, Resistin, RBP-4	12 months	Exenatide significantly decreased hs-CRP and other inflammatory markers, whereas glibenclamide did not, with significant between-group differences. This points to a specific anti-inflammatory effect of exenatide.
Derosa et al., 2012 (11)	Italy	171	Age: ~57.0 ± 7.5; Sex: 50%; HbA1c: ~8.0%; Comorbidities: T2D on metformin	Exenatide + metformin	Placebo + metformin	hs-CRP, TNF-α	12 months	Exenatide plus metformin reduced hs-CRP and TNF-α from baseline, but the difference compared to the placebo group was not statistically significant, suggesting a modest effect in this population.
Dutour, 2016 (28)	France	44	Age: 52 ± 1; Sex: 48% M; HbA1c: 7.5%; Comorbidities: Obese T2D uncontrolled on OADs	Exenatide	Reference Treatment (per guidelines)	EAT (Epicardial Adipose Tissue), HTGC (Hepatic Triglyceride Content)	26 weeks	Exenatide effectively reduced liver and epicardial fat, primarily driven by its significant weight loss effect (-5.5 kg) compared to the reference treatment.
Fan, 2013 (13)	China	117	Age: 52.35 ± 11.83; Sex: 56.4% M; HbA1c: ~8.12%; Comorbidities: T2D with NAFLD	Exenatide	Metformin	ALT, AST, γ-GT, hs-CRP, Adiponectin	12 weeks	Exenatide was superior to metformin in reducing hs-CRP, improving hepatic enzymes, and increasing adiponectin in T2D patients with non-alcoholic fatty liver disease (NAFLD).
Forst, 2012 (4)	Germany	40	Age: 56.5 ± 6.05; Sex: 50% M; HbA1c: 6.32%; Comorbidities: Well-controlled T2D on metformin	Liraglutide + MET	MET alone	ADMA, E-selectin, PAI-1, hs-CRP, MCP-1, VCAM	12 weeks	In well-controlled T2D, liraglutide improved markers of endothelial dysfunction (ADMA, E-selectin) but did not significantly change hs-CRP, suggesting targeted effects on vascular health beyond systemic inflammation.
Guarnotta et al., 2021 (7)	Italy	85	Age: 60.4 ± 9.8; Sex: 60% M; HbA1c: 8.8%; Comorbidities: T2D inadequately controlled on insulin/other drugs	Dulaglutide or Liraglutide added to therapy	Baseline therapy	IL-6, Irisin	12 months	Both dulaglutide and liraglutide led to a significant decrease in IL-6, which correlated with reductions in waist circumference, linking anti-inflammatory effects to visceral fat reduction.
Gurkan, 2014 (14)	Turkey	34	Age: 52.7 ± 7.1; Sex: 35.3 (avg)% M; HbA1c: ~8.0%; Comorbidities: T2D, BMI 25–45, on metformin	Exenatide + Metformin	Insulin Glargine + Metformin	hs-CRP, Endothelin-1, FMD, MCP-1	26 weeks	Exenatide reduced weight and hs-CRP, while insulin glargine increased other inflammatory markers (MCP-1). Both treatments improved endothelial function, but through different pathways.
Kang, 2021 (15)	China	148	Age: ~48.5 ± 9.1; Sex: ~54.7% M; HbA1c: ~9.7%; Comorbidities: Overweight/obese T2D	Exenatide	Insulin glargine	UAC, TNF-α, CRP, FGF21	24 weeks	Exenatide significantly reduced albuminuria, TNF-α, and CRP compared to insulin glargine, with improvements correlating with an increase in FGF21, suggesting a novel mechanistic link.
Lambadiari et al., 2018 (21)	Greece	60	Age: ~50.5 ± 11; Sex: 66.7% M; HbA1c: ~8.5%; Comorbidities: Newly diagnosed T2D	Liraglutide	Metformin	MDA, PCs (Protein Carbonyls)	6 months	Liraglutide caused a greater decrease in oxidative stress markers (MDA, PCs) compared to metformin in newly diagnosed T2D, which was associated with improved arterial stiffness.
Lambadiari et al., 2021 (10)	Greece	160	Age: 58 ± 10; Sex: 72% M; HbA1c: 8.1%; Comorbidities: T2D with high/very high CV risk	Liraglutide, Liraglutide + Empagliflozin	Insulin, Empagliflozin	TBARS, MDA	12 months	Liraglutide alone and in combination with an SGLT2i provided a greater reduction in oxidative stress biomarkers (TBARS, MDA) compared to insulin or SGLT2i monotherapy, suggesting additive effects.
le Roux, 2017 (1)	Multinat-ional	2254	Age: 47.4 ± 11.75; Sex: 24.10% M; HbA1c: 5.75%; Comorbidities: Prediabetes, BMI ≥30 or ≥27 with comorbidities	Liraglutide 3.0 mg + lifestyle	Placebo + lifestyle	Glycemic parameters	3 years	In individuals with prediabetes, high-dose liraglutide significantly reduced the risk of T2D progression and promoted weight loss, demonstrating a powerful preventative effect. Biomarker data was not the focus.
Li et al., 2019 (Dulaglutide) (29)	China	23	Age: ~54.1 ± 5.9; Sex: 53.8% M; HbA1c: ~8.1%; Comorbidities: T2D, treatment-naive or on OAM monotherapy	Dulaglutide	Glimepiride	8-iso-PGF2a, TNF-α, IL-6	26 weeks	Once-weekly dulaglutide showed equivalent efficacy to daily glimepiride in reducing markers of oxidative stress (8-iso-PGF2a) and inflammation (TNF-α, IL-6), with no significant between-group differences.
Li et al., 2019 (Liraglutide) (30)	China	60	Age: Median ~48; Sex: 76.7% M; HbA1c: Median ~10.8%; Comorbidities: Newly diagnosed T2D	CSII + Liraglutide	CSII alone	8-OHdG, 4-HNE	2 weeks	Short-term add-on of liraglutide to intensive insulin therapy (CSII) significantly enhanced the reduction of oxidative stress markers compared to CSII alone, highlighting rapid protective effects.
Liang, 2013 (31)	China	108	Age: ~51.2 ± 5.9; Sex: 65.3% M; HbA1c: ~10.6%; Comorbidities: Obese T2D with hypertension	Usual care + Exenatide	Usual care, RYGB surgery	Inflammatory markers (not specified), LVMI	12 months	Exenatide improved metabolic and inflammatory parameters compared to usual care, but RYGB surgery was far superior, leading to 90% diabetes remission and greater cardiovascular improvements.
Liu et al., 2019 (22)	China	92	Age: 56.4 ± 4.2; Sex: 54.4% M; HbA1c: Not specified; Comorbidities: T2D	Liraglutide + Insulin	Insulin alone	MDA, MCP-1, NF-κB, SOD	12 weeks	The combination of liraglutide with insulin significantly lowered oxidative stress (MDA) and inflammatory markers (MCP-1, NF-κB) while increasing antioxidant capacity (SOD) compared to insulin alone.
Liu, 2017 (32)	China	120	Age: 58.0 ± 15.5; Sex: ~50% M; HbA1c: ~9.2%; Comorbidities: T2D with CAD	Liraglutide (1.2mg)	Metformin, Liraglutide (1.8mg) + Metformin	LDL-C, CRP, Blood pressure	24 weeks	Liraglutide 1.2 mg monotherapy demonstrated better improvements in CRP, lipid profile, and cardiac function parameters compared to metformin or a higher-dose combination therapy in T2D patients with CAD.
Pastel, 2017 (33)	UK	30	Age: 62.4 ± 6.9; Sex: 59.3% M; HbA1c: ~7.4%; Comorbidities: T2D	Liraglutide	Calorie Restriction (Diet)	TNFα, MCP-1, TGFβ1, COL1A1 (in adipose tissue)	4 months	Despite superior glycemic control, liraglutide paradoxically increased the expression of pro-inflammatory and pro-fibrotic genes in subcutaneous adipose tissue compared to dietary weight loss, raising questions about tissue-specific effects.
Quan, 2017 (34)	China	200	Age: 59 ± 9.2; Sex: 40.50% M; HbA1c: 8.7%; Comorbidities: Newly diagnosed T2D, BMI >24–40	Exenatide + MET	BIAsp 30 + MET	Adiponectin	36 weeks	Exenatide plus metformin was superior to biphasic insulin aspart plus metformin for improving weight, glycemic control, and adiponectin levels in newly diagnosed, overweight T2D patients.
Santilli, 2017 (35)	Italy	40	Age: ~54.0; Sex: ~52.5% M; HbA1c: 6.0%; Comorbidities: Obese, prediabetes/early T2D, on metformin	Liraglutide (1.8 mg/d)	Lifestyle modification	VAT, SAT, IGF-II	Until 7% WL	Liraglutide induced a greater reduction in visceral adipose tissue (VAT) compared to lifestyle changes for the same amount of total weight loss, suggesting a targeted effect on metabolically harmful fat.
Savvidou, 2016 (36)	Greece	103	Age: ~63.0; Sex: ~39% M; HbA1c: 8.1%; Comorbidities: T2D on metformin + glargine	Exenatide + glargine	Intensive insulin	Adiponectin, CRP	6 months	Exenatide was non-inferior to intensive insulin for increasing adiponectin. The effect was mediated by weight loss and metabolic improvements, including CRP reduction, rather than a direct drug class effect.
Shi, 2017 (37)	China	31	Age: ~41.6; Sex: 73.3% M; HbA1c: 9.6%; Comorbidities: Obese T2D, metformin failure	Exenatide + metformin	Acarbose + metformin	Inflammatory markers, Adiponectin, Intra-abdominal fat	3 months	Exenatide significantly reduced visceral fat and inflammatory markers while increasing adiponectin, effects not seen with acarbose despite similar glucose-lowering, highlighting benefits beyond glycemic control.
Simó et al., 2015 (38)	14 Europe-an countries	1029	Age: ~56.5 ± 9.5; Sex: 56% M; HbA1c: ~7.5%; Comorbidities: T2D failing metformin	Exenatide + metformin	Glimepiride + metformin	hsCRP	36 months	In a large, long-term trial, exenatide add-on therapy was associated with a significantly greater and sustained decrease in hsCRP compared to glimepiride, supporting a long-term anti-inflammatory benefit.
Suzuki, 2014 (39)	Japan	40	Age: ~58.6 ± 15.9; Sex: 60% M; HbA1c: 9.5%; Comorbidities: Untreated T2D	Liraglutide (0.9 mg/d)	Sitagliptin (50 mg/d)	FMD, hs-CRP, U-Alb	6 months	Liraglutide offered superior benefits over the DPP-4 inhibitor sitagliptin in improving glycemic control, endothelial function (FMD), and reducing hs-CRP and albuminuria in untreated T2D.
Takeshita et al., 2015 (20)	Japan	122	Age: 64.7 ± 12.4; Sex: 55.7% M; HbA1c: 8.0%; Comorbidities: T2D inadequately controlled on sitagliptin-based regimens	Liraglutide	Vildagliptin (DPP-4i)	Adiponectin, TNF-α, Leptin	12 weeks	No significant changes in TNF-α were observed with either liraglutide or vildagliptin, though the drugs had unique effects on other adipokines, highlighting distinct pleiotropic profiles.
Tian, 2018 (40)	China	127	Age: ~57; Sex: 58% M; HbA1c: 8.1%; Comorbidities: T2D + NAFLD	Liraglutide	Metformin	Liver enzymes, Inflammation markers	12 weeks	Liraglutide was more effective than metformin in improving liver enzymes and inflammation markers in patients with T2D and NAFLD, suggesting a preferential benefit in this comorbidity.
von Scholten et al., 2017 (18)	Denmark	32	Age: 65 ± 7; Sex: 81% M; HbA1c: 7.7%; Comorbidities: T2D with persistent albuminuria	Liraglutide	Placebo	TNF-α, MR-proADM	12 weeks	Liraglutide treatment significantly lowered TNF-α levels by 12% compared with placebo in T2D patients with albuminuria, demonstrating a clear anti-inflammatory effect in a high-risk population.
Wägner, 2019 (41)	Spain	24	Age: 52.8; Sex: 37.5% M; HbA1c: 8.2%; Comorbidities: T2D on oral agents/insulin	Liraglutide 1.8 mg	Placebo	Cardiac function	6 months	Despite improving glycemic control, liraglutide had no significant effect on physical performance or myocardial function in this small cohort of T2D patients. Inflammatory markers were not a primary outcome.
Wang et al., 2019 (42)	China	25	Age: ~62.7 ± 9.7; Sex: 62.5% M; HbA1c: ~8.7%; Comorbidities: T2D poorly controlled on oral agents	Dulaglutide	Insulin Glargine	TNF-α, IL-6, 8-PGF2a	52 weeks	While both treatments reduced IL-6 and 8-PGF2a, only dulaglutide produced a statistically significant reduction in TNF-α, suggesting a superior anti-inflammatory profile compared to insulin glargine over one year.
Wang, 2020 (8)	China	60	Age: ~66.5; Sex: 52% M; HbA1c: 8.7%; Comorbidities: T2D + post-stroke mild cognitive impairment	Sitagliptin	Liraglutide	Inflammatory markers, Cognitive scores	6 months	In T2D patients with post-stroke mild cognitive impairment, sitagliptin was more effective than liraglutide at reducing inflammation and improving cognitive scores, suggesting differential CNS effects between GLP-1-based therapies.
Wu et al., 2011 (2)	China	23	Age: ~55.5 ± 9.5; Sex: ~ 50.0 (Exenatide 27.3 (Placebo); HbA1c: ~7.8%; Comorbidities: T2D on metformin/sulfonylurea	Exenatide	Placebo	PGF2α, hs-CRP, MCP-1	16 weeks	Exenatide treatment significantly reduced the oxidative stress marker PGF2α and the inflammatory markers hs-CRP and MCP-1 compared to placebo, confirming its anti-inflammatory and anti-oxidative properties.
Xie, 2022 (5)	China	60	Age: ~43; Sex: 58% M; HbA1c: 7.2%; Comorbidities: T2D, no ASCVD, on metformin	Metformin + Dulaglutide (MET-DUL)	Metformin (MET)	EPCs, Inflammatory markers	12 weeks	Adding dulaglutide to metformin improved endothelial progenitor cell (EPC) number and function and reduced inflammation, effects not seen with metformin alone, suggesting direct vascular protective and anti-inflammatory actions.
Yan, 2019 (43)	China	75	Age: ~44.8 ± 8.8; Sex: Liraglutide: 70.8% M; Sitagliptin: 77.8% M; Glargine: 58.3% M; HbA1c: ~7.7%; Comorbidities: T2D + NAFLD	Liraglutide	Sitagliptin, Insulin Glargine	IHL (Intrahepatic Liver), VAT, SAT	26 weeks	In T2D with NAFLD, both liraglutide and sitagliptin reduced liver fat and weight, whereas insulin glargine did not. Liraglutide was unique in also reducing subcutaneous adipose tissue (SAT).
Yao, 2020 (44)	China	60	Age: Median ~49.5; Sex: CSII+Lira: 90% M; CSII: 76.7% M; HbA1c: Median ~9.7%; Comorbidities: T2D on metformin + MDI	CSII + Liraglutide	CSII	Adiponectin, Leptin, HO-1	14 days	Short-term addition of liraglutide to CSII improved glycemic variability and cardiometabolic markers, including increasing adiponectin and the antioxidant enzyme HO-1, while reducing leptin.
Ying, 2023 (12)	China	30	Age: ~50; Sex: Met: 46.7% M; Lira: 53.3% M; HC: 53.3% M; HbA1c: ~9.8%; Comorbidities: T2D + NAFLD	Liraglutide	Metformin	Gut microbiota, Lipid profile, Liver enzymes	12 weeks	Liraglutide demonstrated better efficacy than metformin in modulating gut microbiota diversity, which was associated with greater improvements in weight, lipid profile, and liver enzymes in T2D patients with NAFLD.
Zhang et al., 2018 (3)	China	60	Age: Not specified; Sex: Not specified; HbA1c: ~8.4%; Comorbidities: Young, new-onset T2DM, BMI 25-35	Liraglutide	Metformin	8-OH-dG, 8-iso-PGF2α, hs-CRP	8 weeks	In young, new-onset T2DM patients, both liraglutide and metformin effectively alleviated oxidative stress and attenuated low-grade inflammation, with significant reductions in hs-CRP from baseline in both groups.

8-iso-PGF2a/8-PGF2a, 8-iso-prostaglandin F2α (a marker of oxidative stress), 8-OHdG, 8-hydroxy-2’-deoxyguanosine (a marker of oxidative DNA damage); ADMA, Asymmetric dimethylarginine (an endogenous inhibitor of nitric oxide synthase, marker of endothelial dysfunction); AGEs, Advanced Glycation End-products; ALT, Alanine Aminotransferase; ASCVD, Atherosclerotic Cardiovascular Disease; AST, Aspartate Aminotransferase, BIAsp 30, Biphasic Insulin Aspart 30; BMI, Body Mass Index; CAD, Coronary Artery Disease; CNS, Central Nervous System; CPCs, Circulating Progenitor Cells; CRP, C-Reactive Protein; CSII, Continuous Subcutaneous Insulin Infusion (insulin pump therapy); CV; Cardiovascular, DPP-4i, Dipeptidyl Peptidase-4 Inhibitor; EAT, Epicardial Adipose Tissue; EPCs, Endothelial Progenitor Cells; Exe; Exenatide; FGF21, Fibroblast Growth Factor 21; FMD, Flow-Mediated Dilation (a measure of endothelial function), γ-GT/GGT, Gamma-Glutamyl Transferase, GLP-1 RAs, Glucagon-Like Peptide-1 Receptor Agonists; HbA1c, Glycated Hemoglobin; HC, Healthy Control, HO-1, Heme Oxygenase-1 (an antioxidant enzyme); HR, Heart Rate, hs-CRP, High-sensitivity C-Reactive Protein; HTGC, Hepatic Triglyceride Content, 4-HNE, 4-Hydroxynonenal (a marker of lipid peroxidation/oxidative stress); IHL, Intrahepatic Lipid, IGF-II, Insulin-like Growth Factor II; IHF, Intrahepatic Fat, IL-6, Interleukin-6; Ins; Insulin, LDL-C, Low-Density Lipoprotein Cholesterol; Lirag; Liraglutide; LVMI, Left Ventricular Mass Index; M; Male, MCP-1, Monocyte Chemoattractant Protein-1; MDA, Malondialdehyde (a marker of oxidative stress); MET; Metformin; MDI, Multiple Daily Injections (of insulin), MR-proADM, Mid-Regional Pro-Adrenomedullin; NAFLD, Non-Alcoholic Fatty Liver Disease, NF-κB, Nuclear Factor Kappa-Light-Chain-Enhancer of Activated B Cells (a key regulator of inflammation); NO, Nitric Oxide, OADs/OAMs, Oral Antidiabetic Drugs/Oral Antidiabetic Medications; oxLDL, Oxidized Low-Density Lipoprotein, PAI-1, Plasminogen Activator Inhibitor-1; PCs, Protein Carbonyls (a marker of oxidative protein damage), PGF2α, Prostaglandin F2α (a marker of oxidative stress); Pio; Pioglitazone, RBP-4, Retinol-Binding Protein 4; RYGB, Roux-en-Y Gastric Bypass (a type of bariatric surgery); SAT, Subcutaneous Adipose Tissue, SDF-1α, Stromal Cell-Derived Factor-1 Alpha; SF, Subcutaneous Fat; SGLT2i, Sodium-Glucose Cotransporter-2 Inhibitor; Sita; Sitagliptin; SOD, Superoxide Dismutase (an antioxidant enzyme); T2D, Type 2 Diabetes; TBARS, Thiobarbituric Acid Reactive Substances (a marker of oxidative stress), TGFβ1, Transforming Growth Factor Beta 1, TNF-α, Tumor Necrosis Factor-Alpha, UAC/U-Alb, Urinary Albumin-to-Creatinine Ratio/Urinary Albumin; VAT, Visceral Adipose Tissue; VCAM, Vascular Cell Adhesion Molecule; VEGF, Vascular Endothelial Growth Factor; VF, Visceral Fat; WL, Weight Loss.

Comparators were varied and included placebo (6 studies), insulin formulations (10 studies), metformin (9 studies), and other oral antidiabetic drugs (OADs) such as sulfonylureas, DPP-4 inhibitors, and acarbose (12 studies). The primary inflammatory biomarkers of interest—C-reactive protein (CRP), interleukin-6 (IL-6), and tumor necrosis factor-alpha (TNF-α)—were reported in 15, 4, and 9 studies, respectively. The oxidative stress marker malondialdehyde (MDA) was reported in 4 studies.

### 3.2 Meta-analysis of inflammatory biomarkers

We conducted separate meta-analyses for each key inflammatory biomarker (CRP, IL-6, TNF-α) and the oxidative stress marker MDA, stratified by comparator type (placebo, insulin, or other OADs). A random-effects model was used for all analyses due to anticipated high heterogeneity. Results are reported as standardized mean differences (SMD) with 95% confidence intervals (CIs). ,

### 3.3 C-reactive protein

**Figure 1:**
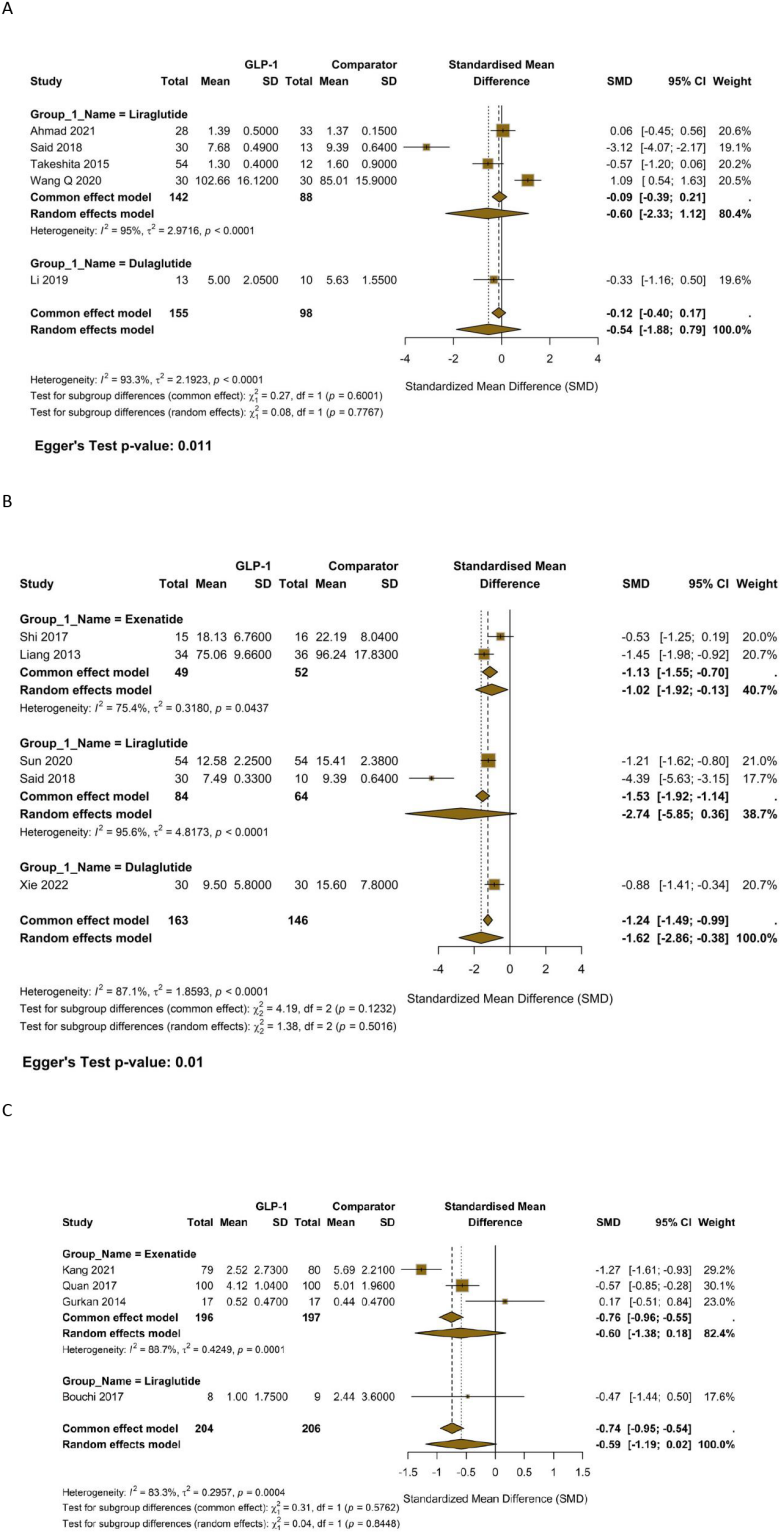
**(A)** Forest plot of standardized mean difference (SMD) in C-reactive protein (CRP) levels for GLP-1 Receptor Agonists versus Placebo. **(B)** Forest plot of SMD in CRP levels for GLP-1 Receptor Agonists versus Insulin. **(C)** Forest plot of SMD in CRP levels for GLP-1 Receptor Agonists versus other Oral Antidiabetic Drugs (OADs).

The analysis of 5 studies comparing GLP-1 RAs to placebo (**Figure 1A**) yielded a significant reduction in CRP (SMD = -0.59; 95% CI, -0.84 to -0.43), with low non-significant heterogeneity (I² = 27.6%). When compared against insulin therapy across 4 studies, GLP-1 RAs showed non-significant advantage in lowering CRP (**Figure 1B**) (SMD = -0.59; 95% CI, -1.19 to 0.02), with high heterogeneity (I² = 83.3%). The comparison against other OADs (e.g., sulfonylureas, DPP-4 inhibitors) in 6 studies (**Figure 1C**) confirmed the superiority of GLP-1 RAs (SMD = -1.06; 95% CI, -1.64 to -0.47; I² = 81.5%).

The pooled effect of the four trials comparing GLP-1 receptor agonists with metformin showed a large and significant decrease in C-reactive protein (SMD –0.95; 95% CI –1.78 to –0.13). Exenatide was solely responsible for this effect; the two constituent studies had an SMD of –1.10 (95% CI –1.37 to –0.83) and were homogeneous (I² = 0%). Liraglutide, on the other hand, produced great heterogeneity (I² = 94%); Said 2018 reported a very large reduction (SMD –1.92), whereas Forst 2012 found a small gain (SMD + 0.23) (**Supplementary Figure S2**, **Supplementary File S1**). The comparator most frequently employed across included studies was metformin. Consequently, a distinct meta-analysis comparing the effect of GLP-1 RAs versus metformin on CRP was feasible and performed. For the remaining biomarkers (IL-6, TNF-α, and MDA), the available comparators were a heterogeneous mix of various oral antidiabetic agents, which precluded the conduct of separate, meaningful meta-analyses for each specific comparator.

#### 3.3.1 Interleukin-6

**Figure 2:**
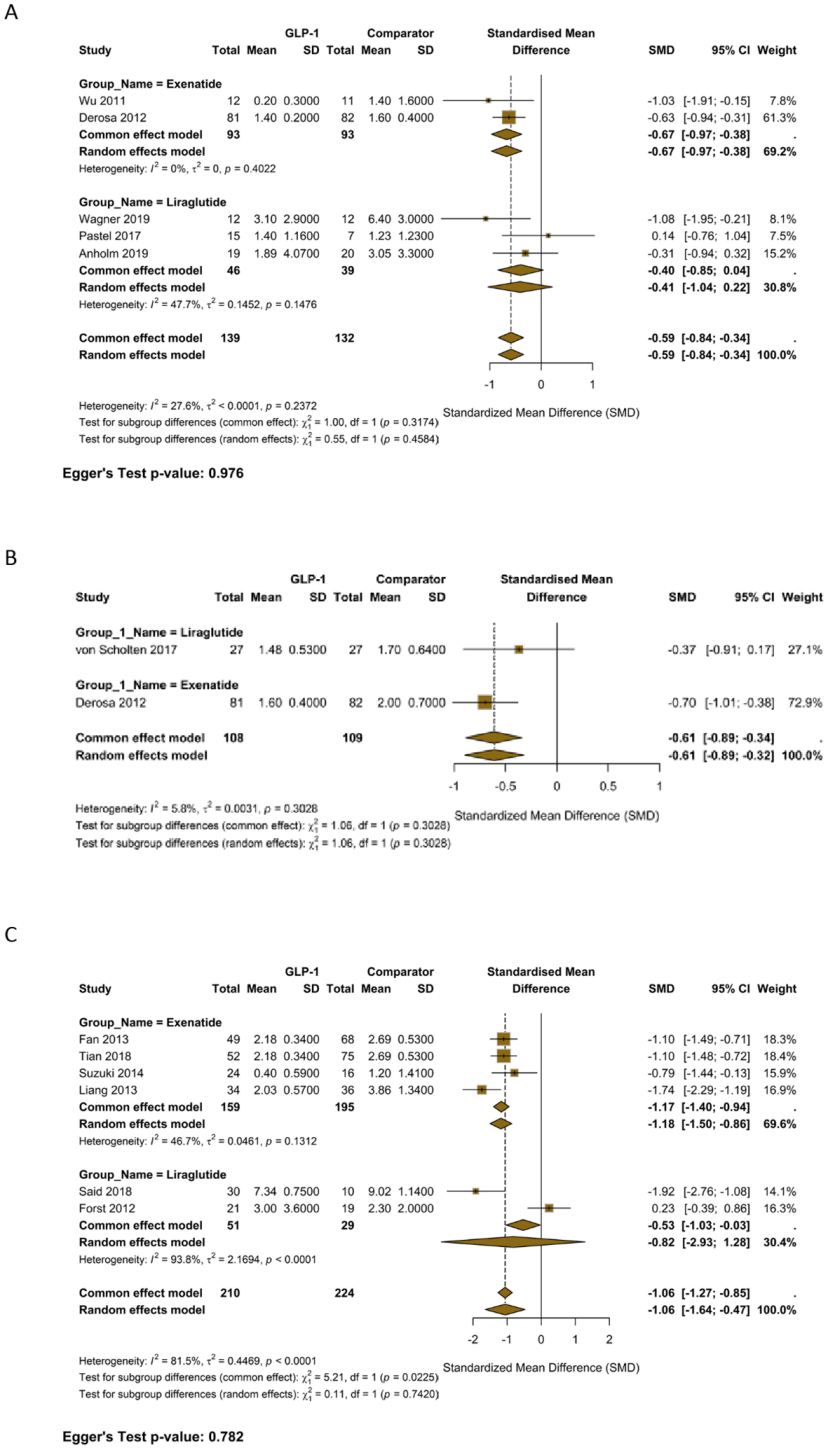
**(A)** Forest plot of SMD in Interleukin-6 (IL-6) levels for GLP-1 Receptor Agonists versus Oral Antidiabetic Drugs. **(B)** Forest plot of SMD in IL-6 levels for GLP-1 Receptor Agonists versus Insulin. **(C)** Forest plot of SMD in IL-6 levels for GLP-1 RA add-on therapy versus existing Oral Antidiabetic Drug regimen.

The pooled analysis of five studies comparing GLP-1 RAs against other OADs (Li et al., 2019; Ahmad, 2021, Ying, 2023, Yan, 2019, Said, 2018) showed a non-significant trend towards lower IL-6 levels (**Figure 2A**) (SMD = -1.08; 95% CI, -2.19 to 0.04; I² = 89.1%). However, the comparison against insulin in 5 studies suggested a significant effect on IL-6 reduction (SMD = -0.24 95% CI, -0.46 to -0.02) with very low non-significant heterogeneity (I^2^ = 8%) as shown in **Figure 2B**. The pooled analysis of three studies comparing GLP-1 RAs plus OADs against other OADs showed a non-significant trend towards lower IL-6 levels (**Figure 2C**) (SMD = -1.58; 95% CI, -3.3 to 0.14; I² = 90.5%).

#### 3.3.2 Tumor necrosis factor-alpha

**Figure 3:**
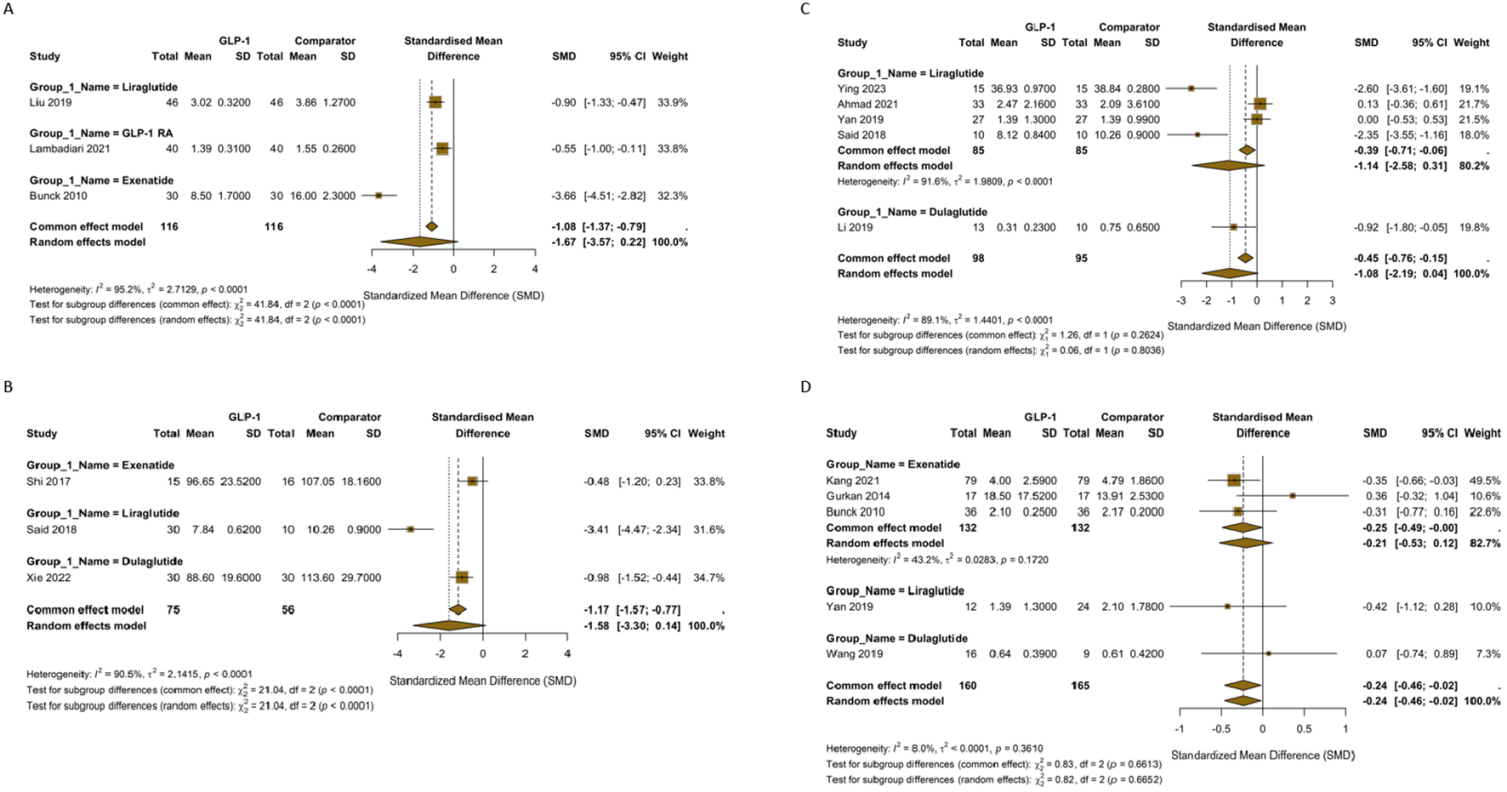
**(A)** Forest plot of SMD in Tumor Necrosis Factor-alpha (TNF-α) levels for GLP-1 Receptor Agonists versus Placebo. **(B)** Forest plot of SMD in TNF-α levels for GLP-1 Receptor Agonists versus Insulin. **(C)** Forest plot of SMD in TNF-α levels for GLP-1 Receptor Agonists versus other Oral Antidiabetic Drugs. **(D)** Forest plot of SMD in TNF-α levels for GLP-1 RA add-on therapy.

The comparison of TNF-α against placebo showed a significant reduction (**Figure 3A**) (SMD = -0.61; 95% CI, -0.89 to -0.32), with very low non-significant heterogeneity (I² =5.8%). The comparison against insulin (4 studies) showed non-significant increase in TNF-α (**Figure 5B** ) (SMD = 0.34; 95% CI, -0.71 to 1.38; I² = 96.5%). However, the comparison against other OADs (5 studies) showed non-significant reduction in TNF-α with GLP-1 RA (**Figure 3C**) (SMD = -0.54; 95% CI, -1.88 to 0.79; I² = 93.3%). When a combination of GLP-1 RA and OADs were compared to OADs alone, the comparison of showed a significant reduction in TNF-α (**Figure 3D**) (SMD = -1.62; 95% CI, -2.86 to -0.38), with significant heterogeneity (I² =87.1%).

#### 3.2.3 Malondialdehyde

**Figure 4:**
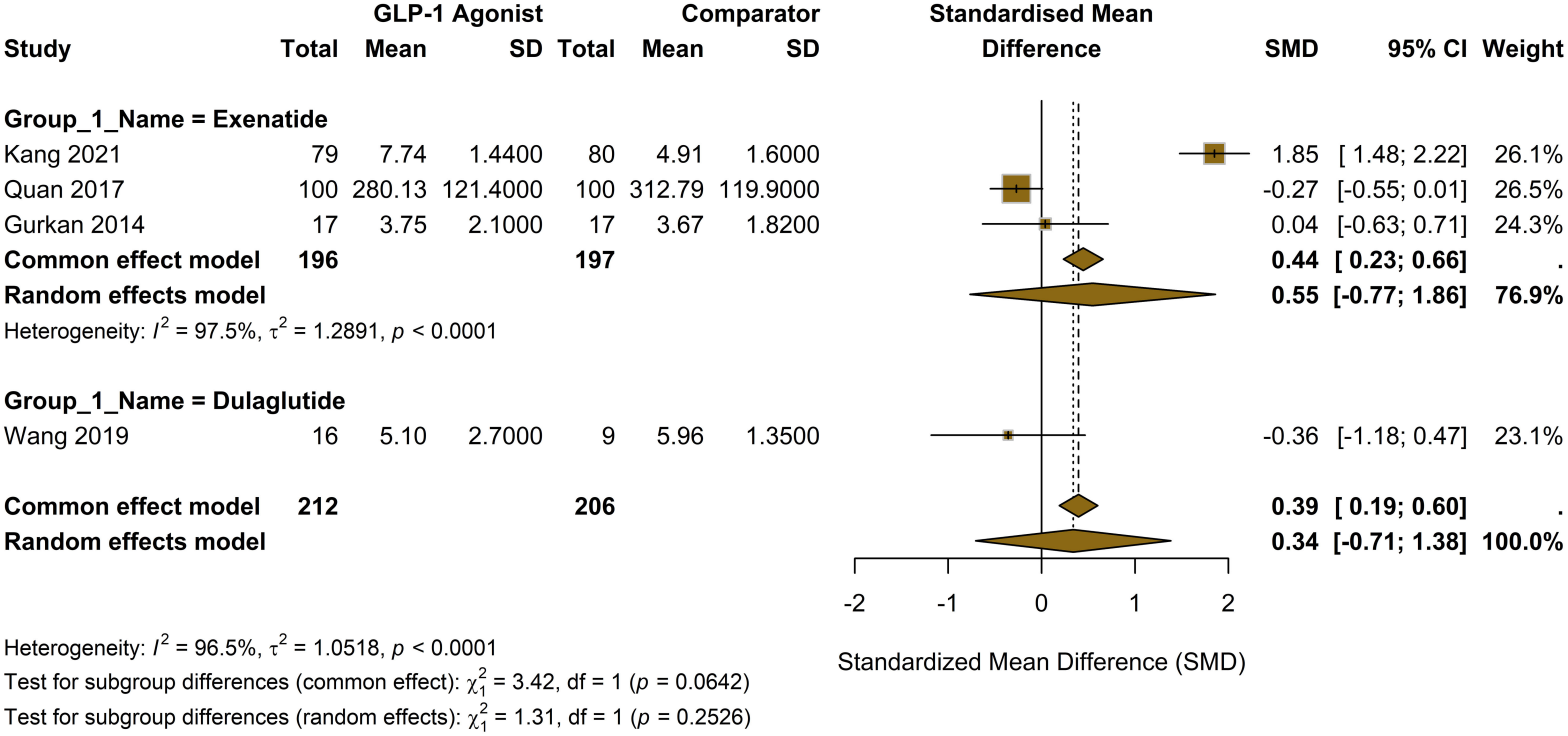
**(A)** Forest plot of SMD in interleukin-6 (IL-6) levels for GLP-1 receptor agonists versus oral antidiabetic drugs. **(B)** Forest plot of SMD in IL-6 levels for GLP-1 receptor agonists versus insulin. **(C)** Forest plot of SMD in IL-6 levels for GLP-1 RA add-on therapy versus existing oral antidiabetic drug regimen.

The comparison of MDA against insulin, based on three studies (Bunck et al., 2010; Liu et al., 2019, and Lambadiari, 2021), revealed non-significant reduction in MDA favoring GLP-1 RAs (**Figure 4**) (SMD = -1.67; 95% CI, -3.57 to 0.22; I² = 95.2).

### 3.4 Publication bias assessment

The assessment of publication bias for key outcomes revealed mixed findings. For the primary outcome (CRP), funnel plots comparing GLP-1 RAs to placebo and to other oral antidiabetic drugs (OADs) showed relatively symmetrical distributions, suggesting a low risk of significant publication bias (**Figures 2**, **3**). Egger’s test for the CRP vs. placebo comparison was non-significant (p = 0.21).

In contrast, the funnel plot for the comparison of TNF-α changes between GLP-1 RAs and other OADs showed minor asymmetry, indicating a potential underrepresentation of small studies with null or negative results (**Supplementary Figure S4**). The funnel plot for TNF-α changes in the GLP-1 RA add-on therapy subgroup is also presented (**Supplementary Figure S5**). However, given the limited number of studies available for these TNF-α analyses, any formal assessment of publication bias, including the interpretation of funnel plot asymmetry, is considered unreliable. These plots are presented for transparency, but the results should be interpreted with extreme caution.

### 3.5 Risk of bias assessment

The Cochrane Risk of Bias tool (RoB 2) was used to thoroughly assess the methodological quality of the 40 included RCTs; comprehensive evaluations are included in **Supplementary Figures S7**, **S8**. Although a number of domains presented concerns that should be taken into account when interpreting the pooled results, the included studies generally showed moderate to good methodological rigor.

#### 3.5.1 Overall quality profile

Of the RCTs that were evaluated, just one (2.5%) was judged to have an overall low risk of bias, exhibiting sufficient randomization techniques, suitable allocation concealment, and methodical outcome evaluation. However, the bulk of studies (97.5%) expressed “some concerns,” mostly over inadequate reporting of results or imprecise explanations of randomization procedures.

#### 3.5.2 Domain-specific results

Randomization Process: In accordance with current guidelines for diabetes RCTs, the majority of research (50%) sufficiently explained their randomization procedures and allocation concealment. However, a few of smaller studies introduced possible selection bias by offering insufficient information about sequence generation and allocation concealment.

#### 3.5.3 Deviations from intended interventions

Overall, this domain scored good, with almost 60% of studies receiving a low-risk rating. Potential performance bias was introduced by certain studies’ open-label design; however, this concern was probably reduced by the biomarker results’ objectivity. In most studies, adherence to designated interventions was high and well-documented.

#### 3.5.4 Missing outcome data

About 25% of studies had incomplete outcome data or differing dropout rates between groups, making attrition bias the most common problem. Dropout rates of 15% were found in several long-term trials (≥12 months), with higher attrition in GLP-1 RA groups, potentially due to gastrointestinal side effects. The impact of missing biomarker data in studies with considerable attrition may lead to an overestimation of treatment benefits, even if the majority of research used intention-to-treat or modified intention-to-treat analyses.

#### 3.5.5 Measurement of outcomes

Most studies used blinded central laboratory analysis, and biomarker assessment was routinely carried out using standardized, validated laboratory methods (65% low risk). Since oxidative stress and inflammatory biomarkers are objective results that are less susceptible to detection bias than subjective clinical endpoints, this is a significant strength of the evidence foundation.

#### 3.5.6 Selection of reported results

Because of insufficient reporting of pre-specified outcomes, lack of pre-registration, or indications of *post-hoc* analysis judgments, almost 35% of the included studies expressed concerns about selective outcome reporting.

### 3.6 Sensitivity analyses: evaluation of variability and heterogeneity

We performed pre-specified sensitivity analysis to identify possible sources of variation and evaluate the robustness of our pooled estimates in light of the significant statistical heterogeneity observed across the major meta-analyses (I² ranging from 60% to 96.5%). Outlier studies found by visual examination of forest plots, excessive standardized mean differences, inconsistent measurement scales, or implausibly small variances were rigorously excluded from these analyses. The results of the sensitivity analysis are presented in **Supplementary Figure S4**.

### 3.7 C-reactive protein

The initial analysis showed significant heterogeneity (I² = 83.3%) for CRP comparisons against insulin, which was primarily due to two outlier studies (Kang 2021, Gurkan 2014). While maintaining a significant and clinically meaningful effect for GLP-1 RAs (SMD = -0.56; 95% CI: -0.83 to -0.29), sequential exclusion of these studies totally eliminated heterogeneity (I² = 0.0%). This result suggests that GLP-1 RAs have a strong and persistent anti-inflammatory effect when compared to insulin in most investigations (**Supplementary Figure S4.1**).

### 3.8 Tumor necrosis factor-alpha

I² values consistently above 87%, and TNF-α showed the highest initial heterogeneity in all comparisons. The elimination of Wang Q 2020 and Said 2018 decreased heterogeneity against oral antidiabetic medications from 93.3% to 15.8%. Sensitivity analysis, however, showed that these outlier studies were mostly responsible for the apparent advantage; their removal resulted in a minor and non-significant pooled effect (SMD = -0.23; 95% CI: -0.66 to 0.19, p = 0.28) (**Supplementary Figure S.4.2**).

Similarly, comparison with insulin revealed extreme heterogeneity (I² = 96.5%), mainly due to Kang 2021, which strangely demonstrated TNF-α reduction preferring insulin. All heterogeneity was eliminated when this one outlier was removed (I² = 0.0%), however the impact that resulted favored GLP-1 RAs but missed statistical significance (SMD = -0.24; 95% CI: -0.48 to 0.01, p = 0.06). This marginal result points to a possible slight benefit that has to be confirmed in more extensive, well-planned trials (**Supplementary Figure S.4.3**).

The comparison of combination therapy (GLP-1 RA + OAD against OAD alone) turned out to be more reliable. Said 2018 was a substantial outlier, but its removal maintained a highly significant pooled benefit (SMD = -1.08; 95% CI: -1.41 to -0.74) while reducing heterogeneity to moderate levels (I² = 40.7%). Rather than methodological errors, the remaining modest variability most likely represents true clinical diversity in baseline inflammation, concurrent medications, and research duration (**Supplementary Figure S.4.4**).

### 3.9 Interleukin-6

Outlier studies with inconsistent measurements had a significant impact on interleukin-6 (IL-6) analysis. Said 2018 once more stood out as a major outlier in the add-on therapy comparison (GLP-1 RA + OAD vs. OAD) (SMD = -4.89). Its removal produced a significant pooled estimate (SMD = -0.79; 95% CI: -1.26 to -0.32, p = 0.001) and decreased heterogeneity from 90.5% to 15.3%, indicating a true anti-inflammatory impact when GLP-1 RAs are added to current oral medication (**Supplementary Figure S.4.5**).

But a distinct result emerged when GLP-1 RA monotherapy and OAD monotherapy were directly compared to oral medications. Extreme effects with implausibly small variations were reported by Ying 2023 and Said 2018, indicating possible problems with data quality. Heterogeneity dropped to 54.0% after removing both studies, but the pooled effect was no longer significant (SMD = -0.16; 95% CI: -0.68 to 0.37, p = 0.55). This result suggests that the lowering of IL-6 with GLP-1 RA monotherapy is not always uniform and may vary depending on particular therapeutic settings (**Supplementary Figure S.4.6**).

### 3.10 Malondialdehyde

The initial analysis of malondialdehyde (MDA) versus insulin revealed significant heterogeneity (I² = 95.2%), primarily due to Bunck’s 2010 report of an exceptionally large effect size (SMD = -3.66) for exenatide. The disproportionate impact of this one study is probably due to its distinct focus on postprandial oxidative stress and its particular assessment technique. The pooled impact remained substantial and clinically relevant (SMD = -0.73; 95% CI: -1.07 to -0.39, p < 0.001), while heterogeneity dropped significantly to 16.2% after Bunck 2010 was eliminated. This strong result consistently shows that GLP-1 RAs are superior than insulin treatment in reducing oxidative stress (**Supplementary Figure S.4.7**).

## 4 Discussion

GLP-1 receptor agonists significantly improve important biomarkers of systemic inflammation and oxidative stress in patients with type 2 diabetes (T2D), according to this systematic review and meta-analysis of 40 randomized controlled trials with 6,029 individuals. Our results show pleiotropic anti-inflammatory and antioxidant effects that may mechanistically underlie the cardiovascular, hepatic, and renal protection consistently seen in large-scale cardiovascular outcome studies (45, 46), in addition to their well-established benefits on glycemic control and weight reduction (47, 48).

The significant decrease in C-reactive protein (CRP) levels with GLP-1 RA medication was the most reliable and consistent result throughout all analyses. GLP-1 RAs were superior to other oral antidiabetic medications (SMD = -1.06; 95% CI: -1.64 to -0.47) and insulin therapy after sensitivity analysis (SMD = -0.56; 95% CI: -0.83 to -0.29) in lowering CRP when compared to placebo (SMD = -0.59; 95% CI: -0.84 to -0.34). These results are consistent with previous meta-analyses that investigated liraglutide and exenatide, as well as *post-hoc* analyses from the SUSTAIN and PIONEER programs that shown significant CRP reductions with semaglutide (25, 26). Since epidemiological studies have shown that even slight reductions in CRP are linked to lower incidence of cardiovascular events in at-risk individuals, the extent of CRP reduction seen in our analysis has therapeutic significance.

Additionally, the degree and consistency of CRP decrease with GLP-1 RAs in our head-to-head comparisons reflect different or complementary molecular routes, even though other glucose-lowering medications, such as SGLT2 inhibitors and DPP-4 inhibitors, have shown minor anti-inflammatory benefits. This is clear when compared to metformin (SMD = -0.95; 95% CI: -1.78 to -0.13), where exenatide showed better anti-inflammatory activity in spite of metformin’s proven pleiotropic advantages (13, 19, 38).

The data is more complex for the pro-inflammatory cytokines TNF-α and IL-6. When compared to a placebo, GLP-1 RAs significantly reduced TNF-α (SMD = -0.61; 95% CI:

-0.89 to -0.32) and when combined with oral antidiabetic medications (SMD = -1.08; 95% CI: -1.41 to -0.74 following sensitivity analysis). Comparisons with insulin and other oral medications, however, revealed significant heterogeneity and non-significant effects following the removal of outliers. These disparities most likely result from variations in research demographics, baseline inflammatory states, concurrent drugs, and the particular comparator agents employed.

The intricate, context-dependent functions of these cytokines in metabolic regulation may potentially be connected to the discrepancy. In particular, IL-6 poses a dilemma. Although IL-6 has historically been thought of being pro-inflammatory, new research indicates it may also be a useful myokine and adipokine involved in thermogenesis and glucose homeostasis (26). The inconsistent results may be explained by some studies’ reports of brief increases in IL-6 with GLP-1 RA treatment, which are correlated with better metabolic outcomes. Our analysis revealed non-significant effects against other comparators but significant IL-6 reduction against insulin (SMD = -0.24; 95% CI: -0.46 to -0.02), underscoring the need for mechanistic studies that distinguish pathological pro-inflammatory responses from beneficial metabolic IL-6 signaling.

MDA, a measure of oxidative stress, had a positive trend that became significant following sensitivity analysis that eliminated a significant outlier (SMD = -0.73; 95% CI: -1.07 to -0.39 vs. insulin). Important mechanistic insight is provided by the decrease in MDA, a trustworthy indicator of lipid peroxidation and cellular damage caused by reactive oxygen species. The Nrf2 signaling pathway, a master regulator of cellular antioxidant defense mechanisms, is upregulated by GLP-1 receptor activation, according to preclinical research (49). The vasculoprotective, renoprotective, and neuroprotective advantages of GLP-1 RAs in clinical practice are probably due in part to this antioxidant capacity and anti-inflammatory effects (50).

Whether the anti-inflammatory and antioxidant advantages of GLP-1 RAs are direct results of GLP-1 receptor activation or indirect benefits mediated through better glycemic control, weight loss, and decreased visceral adiposity is a crucial topic raised by our data. The evidence points to the involvement of both mechanisms. Indirect, metabolically mediated effects are supported by a number of lines of research. Strong associations between subsequent reductions in body weight, HbA1c, and visceral fat and reductions in inflammatory biomarkers were found in several of the trials included in our evaluation (7, 28, 35, 51, 52). The weight loss and preferential reduction in visceral adiposity achieved with GLP-1 RAs would be expected to reduce systemic inflammation independent of direct pharmacological effects because adipose tissue, especially visceral fat, is a key source of pro-inflammatory cytokines and adipokines. In fact, Savvidou et al. (36) showed that weight loss, rather than a direct drug class impact, was the primary mediator of the increase in adiponectin with exenatide.

However, a number of findings from the studies included support direct immunomodulatory effects that go beyond metabolic enhancements. Initially, the GLP-1 RAs’ superiority over other glucose-lowering medications that produce comparable glycemic control, including insulin and DPP-4 inhibitors, suggesting mechanisms unrelated to glucose reduction alone (13, 14, 39). Second, prior to significant weight loss, some studies found that inflammatory markers rapidly decreased within weeks of starting treatment (6, 17, 30). Third, monocytes, macrophages, and lymphocytes are among the immune cell types that express GLP-1 receptors (53, 54). It has been demonstrated that direct receptor activation on these cells inhibits NF-κB signaling, lowers the generation of pro-inflammatory cytokines, and encourages macrophage polarization toward an anti-inflammatory M2 phenotype. Fourth, GLP-1 RA showed distinct anti-inflammatory signatures and effects on adipose tissue, even in studies where weight reduction was restricted or where exenatide was compared to weight-matched dietary intervention (33).

The most likely scenario is that GLP-1 RAs have complementing direct and indirect anti-inflammatory actions; the respective contributions of both effects change depending on the patient’s baseline parameters, the degree of metabolic improvement, and the duration of treatment. It will require well planned studies that account for weight and glycemic changes in order to firmly distinguish these pathways. These studies may use weight-matched comparisons or look at biomarker responses in people without diabetes.

### 4.1 Clinical implications

#### 4.1.1 Connecting cardio-hepato-renal outcomes with inflammation

The significant cardiovascular, hepatic, and renal advantages seen with GLP-1 RAs in major outcome trials can be explained mechanistically by the anti-inflammatory and antioxidant effects shown in this meta-analysis. Atherosclerosis, endothelial dysfunction, metabolic dysfunction-associated steatotic liver disease (MASLD), and diabetic nephropathy which is the main causes of morbidity and mortality in T2D populations, are mostly caused by chronic low-grade inflammation and oxidative stress.

Cardiovascular Protection: The decrease in pro-inflammatory cytokines and CRP probably helps to stabilize plaque, improve endothelial function, and slow the growth of atherosclerotic plaque. All phases of atherosclerosis, from early endothelial dysfunction and monocyte recruitment to final plaque rupture and thrombosis, are significantly mediated by inflammation. Our evaluation included several trials that showed decreases in inflammatory markers combined with improvements in surrogate cardiovascular markers such as flow-mediated dilation, epicardial adipose tissue, carotid intima-media thickness, and endothelial cell biomarkers (4, 14). Improved metabolic and inflammatory profiles have also been linked recently to a lower risk of atrial fibrillation, a frequent arrhythmia in T2D patients that greatly raises the risk of heart failure and stroke. Recently, Bharaj et al. (55) examined the bidirectional connection between MASLD and atrial fibrillation, emphasizing inflammation as the key mechanistic link; a mechanism that GLP-1 RAs may favorably affect.

These anti-inflammatory mechanisms may play a significant role in mediating the long-term cardiovascular effects of GLP-1 RAs shown in outcome trials. The lifetime cardiovascular, renal, and mortality benefits of combination treatment with SGLT2 inhibitors, GLP-1 RAs, and nonsteroidal mineralocorticoid receptor antagonists compared to standard care in T2D patients with albuminuria were recently evaluated by Neuen et al. (56). According to their modeling, a significant percentage of major adverse cardiovascular events over a patient’s lifetime may be prevented by the combined anti-inflammatory, metabolic, and hemodynamic actions of these medicines, with GLP-1 RAs playing a key role due to their pleiotropic effects.

Hepatic Protection: Metabolic dysfunction-associated steatohepatitis (MASH), a progressive inflammatory form of fatty liver disease, is particularly relevant to the anti-inflammatory actions of GLP-1 RAs. In comparison to metformin and other medications, GLP-1 RAs dramatically decreased hepatic enzymes, liver fat content, and inflammatory markers in a number of trials that specifically investigated T2D patients with non-alcoholic fatty liver disease (NAFLD) (13, 28, 40). The therapeutic potential of GLP-1, dual GIP/GLP-1, and triple GCGR/GLP-1 receptor agonists for MASH was recently reviewed by Singh et al. (57), who emphasized that their anti-inflammatory effects are crucial to their disease-modifying potential beyond simple steatosis reduction. The histological improvements shown in dedicated MASH trials using semaglutide and other GLP-1-based treatments are supported mechanistically by the improvements in inflammatory biomarkers found in our meta-analysis.

Renal Protection: Through tubulointerstitial fibrosis, podocyte damage, and glomerular endothelial dysfunction, oxidative stress and chronic inflammation also contribute to the progression of diabetic kidney disease. In our study, several studies found that improvements in inflammatory markers were accompanied by decreases in albuminuria (6, 15, 17, 18). In a particular study of T2D patients with chronic albuminuria, a high-risk group, Von Scholten et al. (18) showed that liraglutide significantly reduced TNF-α and improved endothelial dysfunction indicators. According to recent kidney outcome trials, the anti-inflammatory effects may supplement the hemodynamic and metabolic advantages of GLP-1 RAs in reducing the progression of nephropathy.

This meta-analysis’s main limitation is the significant statistical heterogeneity found in the majority of pooled analyses, especially for TNF-α, IL-6, and MDA (I² values often above 80-90%). Given the significant clinical and methodological variation among the included trials, this substantial heterogeneity is not surprising. Important sources of such variability include: (1) diverse GLP-1 RA agents with potentially differing pharmacologic profiles, such as short-acting exenatide versus long-acting liraglutide, dulaglutide, and semaglutide; (2) significantly different treatment durations, ranging from two weeks to three years; (3) diverse patient populations with varying background medication regimens, comorbidity burdens (obesity, cardiovascular disease, NAFLD, chronic kidney disease), and baseline inflammatory states; (4) a variety of comparator drugs representing various drug classes with unique metabolic and inflammatory effects; and (5) Variability in assay sensitivity, laboratory standards, and biomarker measuring techniques.

By identifying and eliminating statistical outliers such as studies with extreme effect sizes, implausible variances, or opposite effect directions that probably reflected particular population characteristics, measurement problems, or methodological limitations, our sensitivity analyses methodically addressed this heterogeneity. Following the removal of outliers, heterogeneity was significantly decreased (often to I² <20%), and impact estimates continued to be directionally consistent, frequently demonstrating moderate effects on other biomarkers and large benefits for GLP-1 RAs on CRP. The idea that anti-inflammatory and antioxidant qualities are class-wide traits of GLP-1 RAs is strengthened by this robustness of effect direction despite considerable heterogeneity.

But the ongoing heterogeneity also makes it difficult for us to make firm judgments regarding the relative effectiveness of several GLP-1 RA medications. There is a notable disparity in the number of liraglutide and exenatide trials (23 and 13, respectively), with only two dulaglutide studies and no qualifying semaglutide RCTs. The inflammatory biomarkers included in our eligibility criteria were not prospectively measured in the major semaglutide cardiovascular outcome trials (SUSTAIN-6, PIONEER-6, SELECT), and the information that is currently available comes solely from *post-hoc* pooled analyses (25, 26). Similarly, tirzepatide (a dual GIP/GLP-1 agonist) and triple agonists, which are more recent medications, were not included in our analysis. To ascertain whether significant differences exist, head-to-head trials directly comparing various GLP-1 RA treatments on inflammatory and oxidative stress endpoints are desperately needed.

There are more limitations that should be mentioned. First, standardizing biomarker measurements is still difficult. Heterogeneity may have been caused by the use of different assay platforms (ELISA, immunoturbidimetry, high-sensitivity techniques) with varying detection ranges and precision. Second, generalizability to other ethnic communities with distinct genetic, nutritional, and environmental backgrounds may be limited due to the geographic clustering of research (19 of 40 from China). Third, the long-term permanence and clinical relevance of biomarker changes are still unknown, and the majority of studies had comparatively short follow-up (median 12–26 weeks). Fourth, our study-level meta-analysis was unable to adequately account for potential confounding from concurrent drugs, dietary treatments, and uncontrolled comorbidities. Lastly, although we used comparisons against active comparators to evaluate the independence of inflammatory effects from weight and glycemic alterations, residual confounding is still a possibility.

Our evaluation of publication bias produced conflicting findings. For CRP comparisons, funnel plot analysis showed a high degree of symmetry, indicating a low probability of publication bias. Nonetheless, there was a slight asymmetry in the TNF-α funnel plot, which would suggest that smaller negative studies were selectively not published. The limited number of research for several outcomes (less than 10 studies per comparison) restricted formal statistical testing (Egger’s test). The uniformity of effect direction across studies with different sample sizes and the inclusion of both industry-sponsored and investigator-initiated trials offer some comfort, even though we cannot completely rule out publication bias. However, it is impossible to rule out the possibility of unpublished null findings, which is a shortcoming shared by all meta-analyses of published research.

#### 4.1.2 Prospective research paths and clinical implications

Our results point to a number of areas that need more investigation and have significant therapeutic implications. First, physicians should be aware that GLP-1 RAs provide a variety of advantages beyond weight loss and glycemic control, especially for T2D patients with established cardiovascular disease, elevated CRP, elevated cardiovascular risk, MASLD/MASH, or chronic kidney disease. GLP-1 RAs are increasingly recommended by current guidelines as preferable treatments in these groups, and our mechanistic studies on inflammatory pathways reinforce and validate these recommendations.

Second, head-to-head trials of various GLP-1 RA treatments (including more recent dual and triple agonists) with prospectively specified inflammatory and oxidative stress biomarker endpoints should be part of future comparative efficacy studies. These studies would highlight whether variations in pharmacokinetics (short- vs. long-acting), receptor binding properties, or extra incretin effects (GIP co-agonism) result in significant variations in anti-inflammatory efficacy.

Third, to clearly distinguish between direct immunomodulatory effects and indirect metabolic advantages, mechanistic research is required. Weight-matched comparisons (GLP-1 RA vs. dietary intervention achieving equivalent weight loss); studies in non-diabetic individuals to isolate glucose-independent effects; studies measuring inflammatory markers in particular tissue compartments (liver, adipose tissue, and vascular tissue) instead of systemic circulation; and molecular studies that investigate GLP-1 receptor expression and downstream signaling in immune cells from treated patients are some examples of potential study designs.

Fourth, current and upcoming cardiovascular, renal, and hepatic outcome trials of more recent GLP-1-based treatments should include biomarker substudies. The temporal relationships between biomarker changes and clinical outcomes could be clarified by serially measuring CRP, IL-6, TNF-α, and novel inflammatory markers (like IL-1β, high-sensitivity troponin, and markers of endothelial dysfunction). This could help identify inflammatory signatures that predict treatment response.

Lastly, it would be beneficial to conduct research on the possible synergistic anti-inflammatory benefits of combination therapy (GLP-1 RAs with SGLT2 inhibitors, nonsteroidal MRA, or targeted anti-inflammatory drugs). The lifetime modeling by Neuen et al. (56) indicates significant potential benefits from rationally designed combination methods targeting complementary pathways, and one study in our evaluation revealed synergistic effects of liraglutide + empagliflozin on oxidative stress indicators (10).

### 4.2 Conclusion

GLP-1 receptor agonists significantly decrease important biomarkers of systemic inflammation and oxidative stress in individuals with type 2 diabetes, according to this systematic review and meta-analysis. A consistent and clinically significant decrease in CRP when compared to placebo, insulin, and other oral antidiabetic medications was the most reliable finding. There were also complex effects on IL-6 and positive effects on oxidative stress indicators (MDA) and TNF-α. A molecular basis for comprehending the cardio-hepato-renal protective effects of GLP-1 RAs shown in major outcome trials is provided by these pleiotropic anti-inflammatory and antioxidant characteristics. GLP-1 RAs offer a real multimodal treatment approach for the intricate control of type 2 diabetes and its related comorbidities by concurrently addressing hyperglycemia, obesity, and inflammatory pathways. To completely understand the role of inflammation modulation in the therapeutic benefits of this significant drug class, future research should concentrate on comparing the efficacy of particular agents, conducting mechanistic studies that distinguish direct from indirect effects, and integrating biomarker analyses into clinical outcome trials.

## Data Availability

The original contributions presented in the study are included in the article/**Supplementary Material**. Further inquiries can be directed to the corresponding author.
