# Supplementary material for: Inflammatory biomarker response to GLP-1 receptor agonists versus other glucose-lowering medications in patients with type 2 diabetes: a systematic review and meta-analysis
Source: Front Endocrinol (Lausanne). 2026 Jan 15;16:1734549. doi: 10.3389/fendo.2025.1734549 (PMC12852008; doi:10.3389/fendo.2025.1734549)
Supplement: Supplementary file 1 [file DataSheet1.docx]

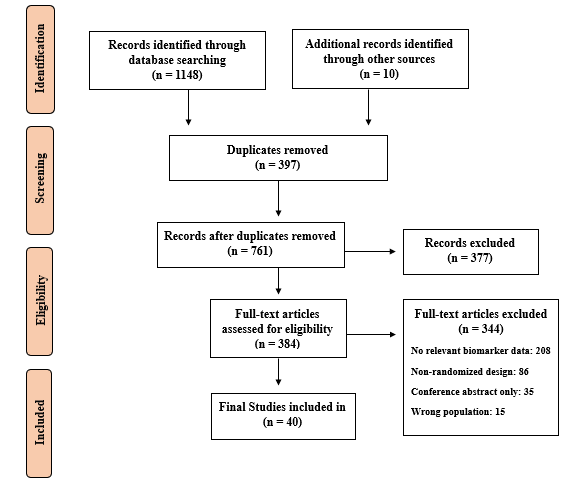


**Figure S1:** PRISMA Flow Diagram for Systematic Reviews


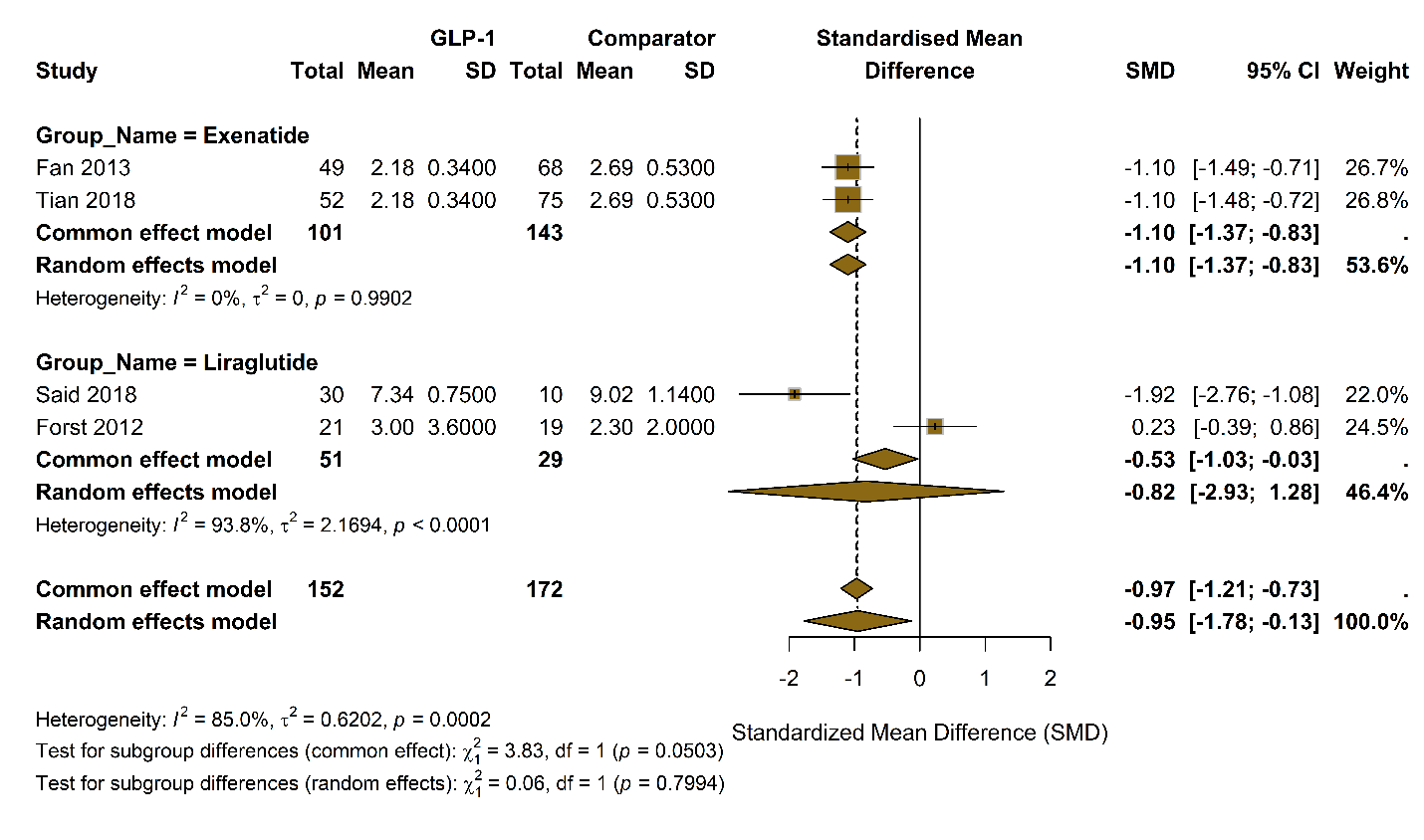


**Figure S2: Forest plot of standardized mean difference (SMD) in C-reactive protein (CRP) levels for GLP-1 Receptor Agonists versus metformin.**


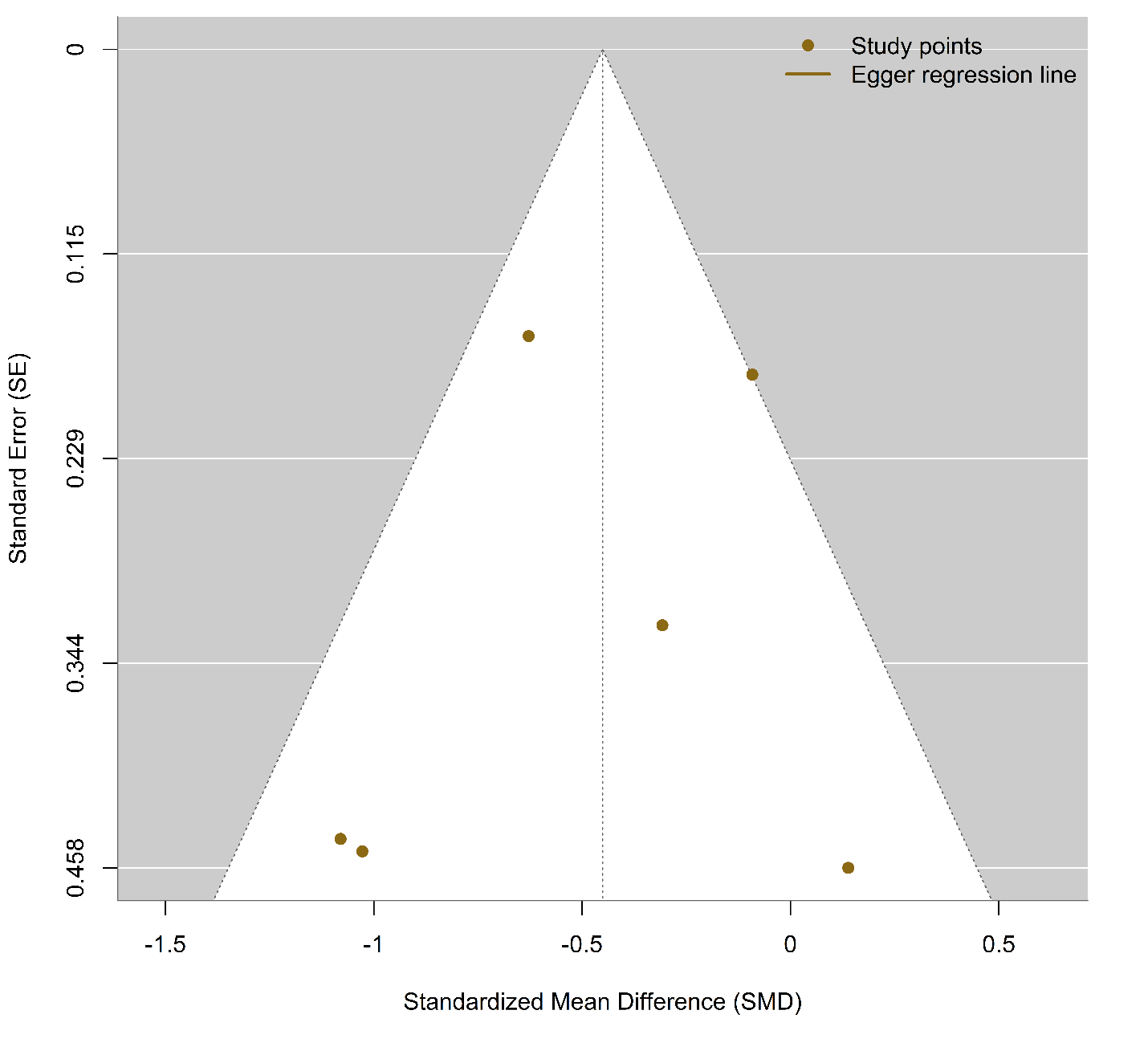


**Figure S3:** Funnel Plot CRP GLP1 Vs Oral Antidiabetic Drug


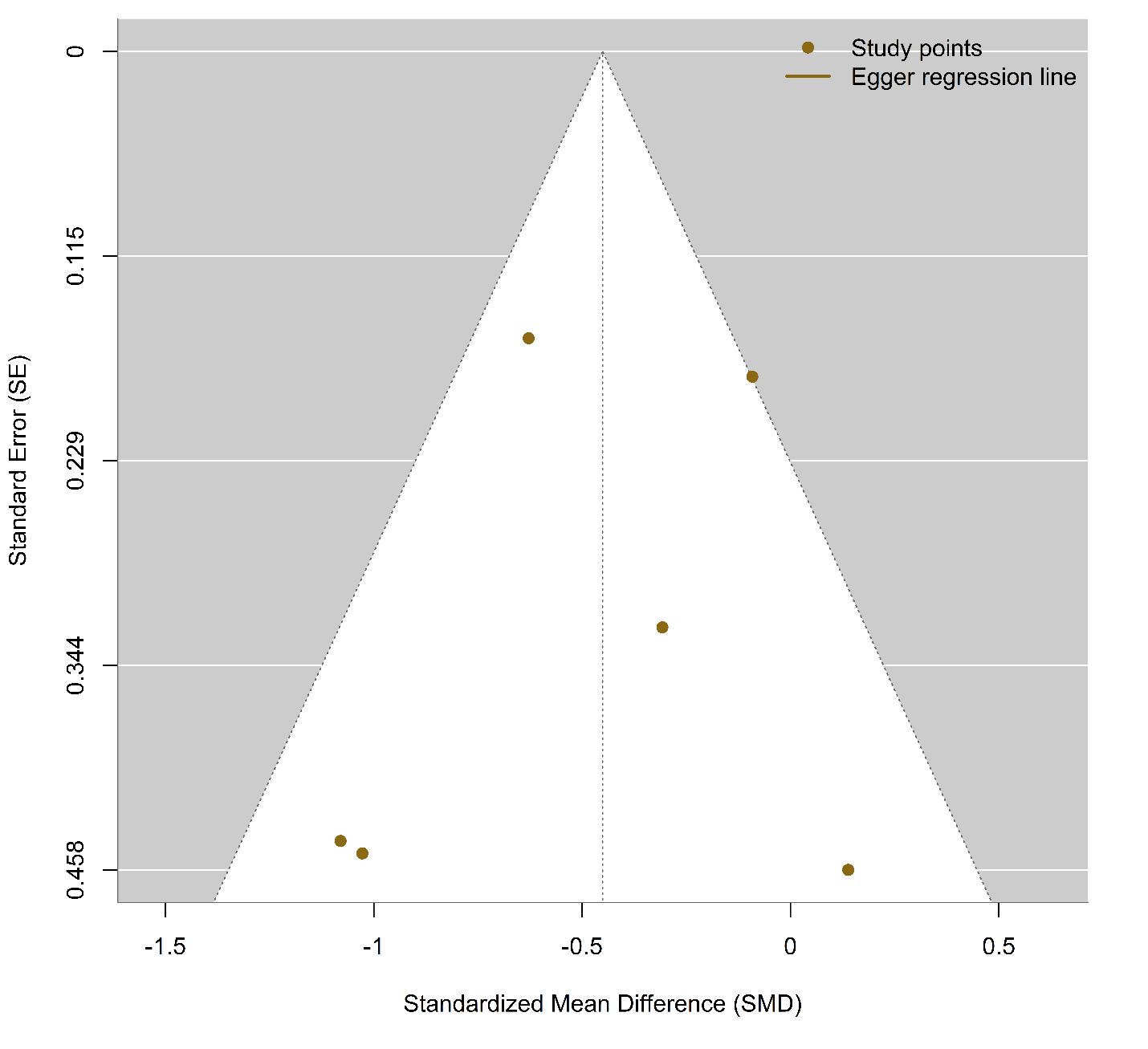


**Figure S4:** Funnel Plot CRP GLP1 Vs Placebo


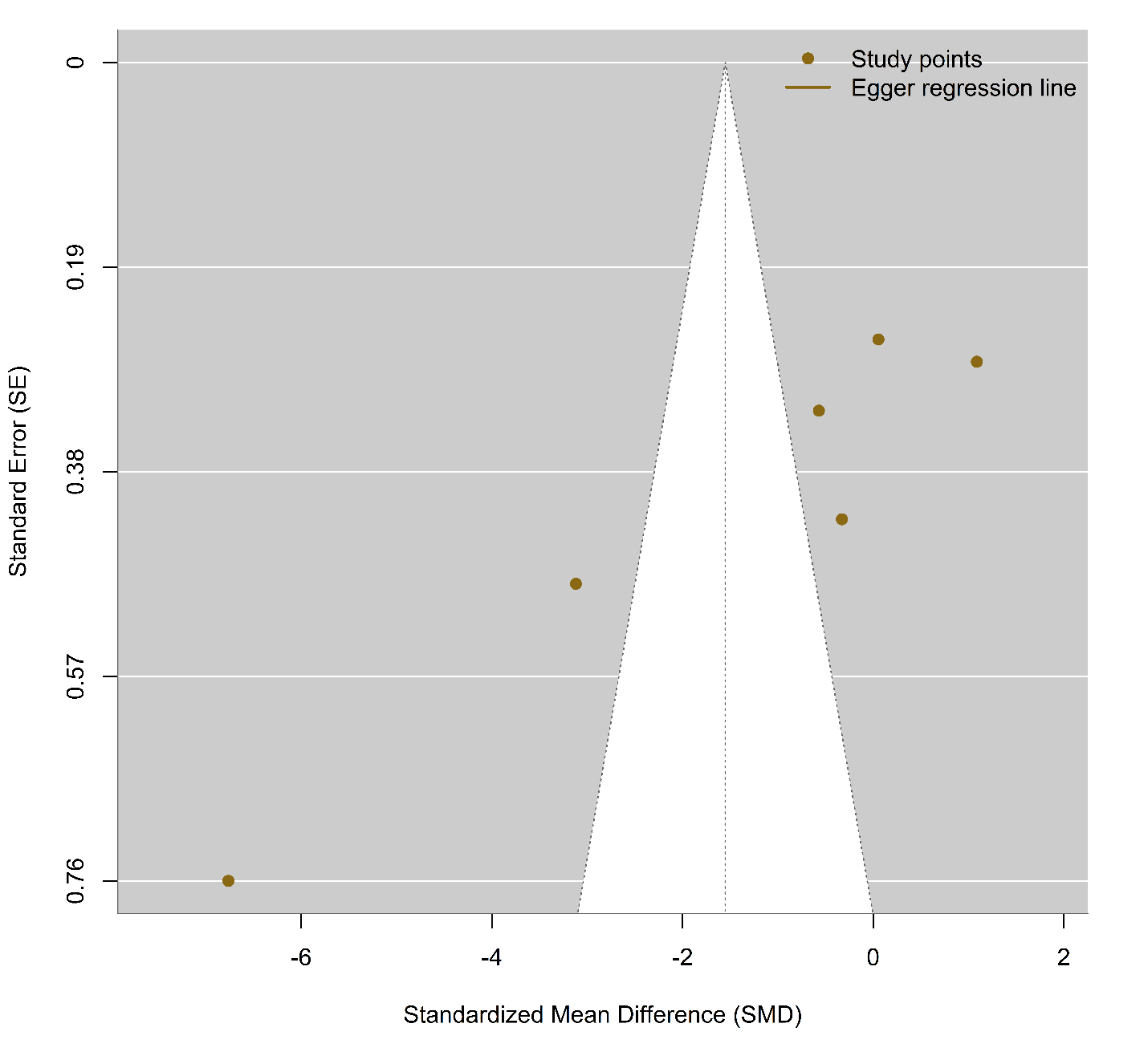


**Figure S5:** Funnel Plot TNFa GLP1 Vs Oral Antidiabetic Drug


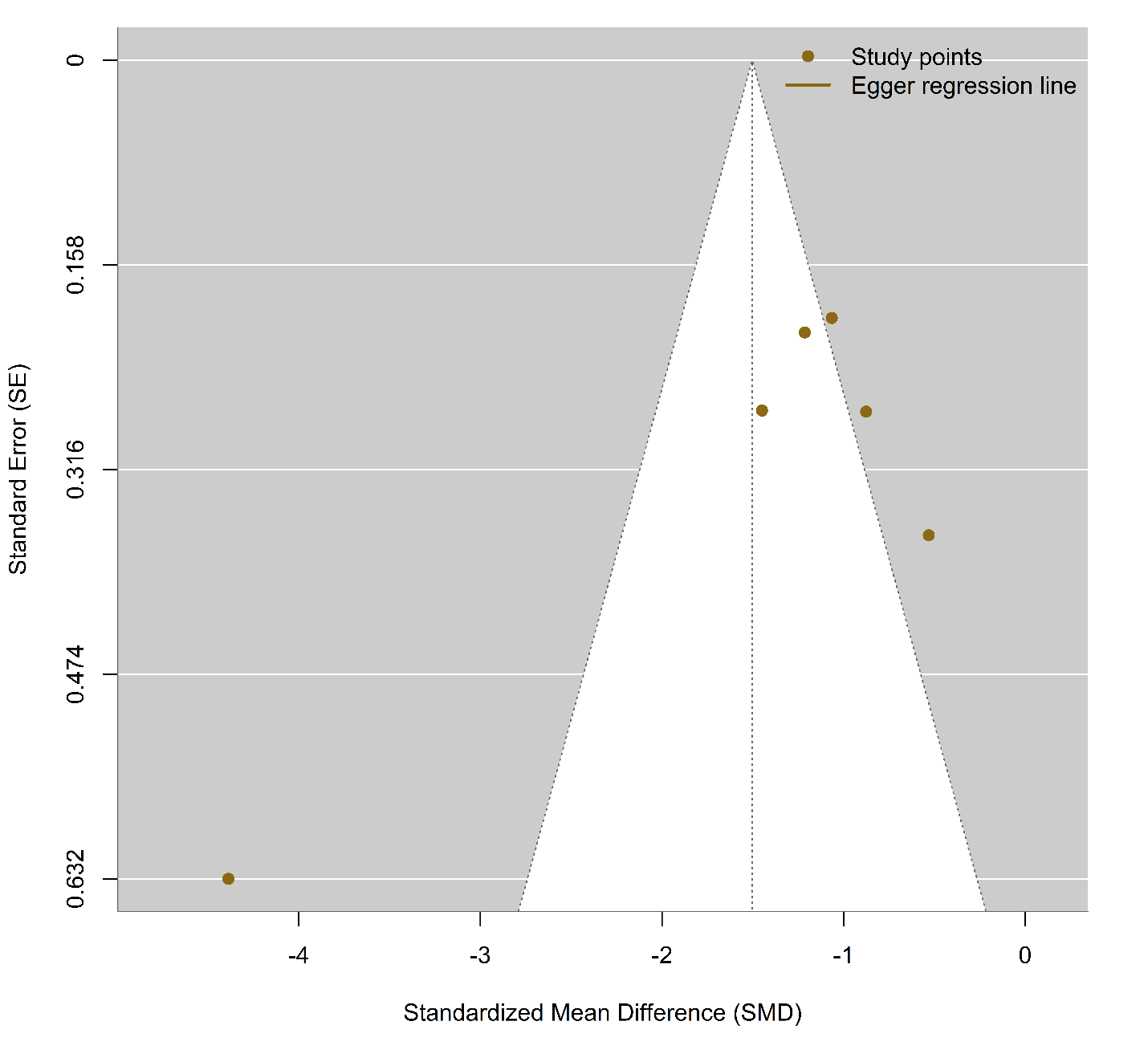


**Figure S6:** Funnel Plot TNFa GLP 1 RA + Oral Antidiabetic Drug Vs Oral Antidiabetic Drug


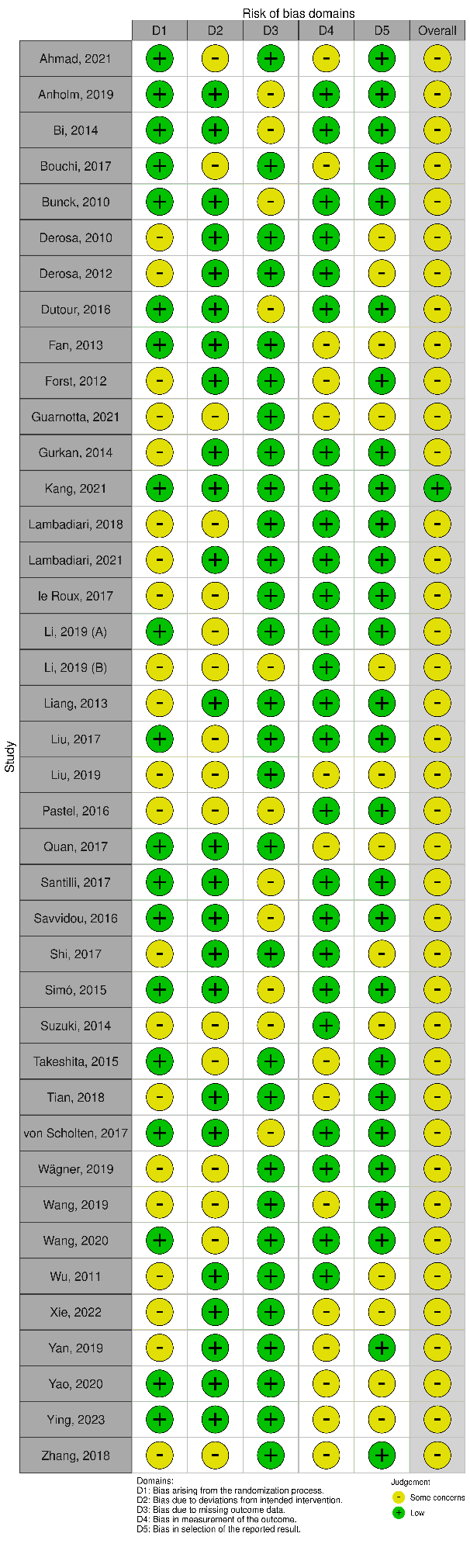
**Figure S7:** Risk of Bias Graph


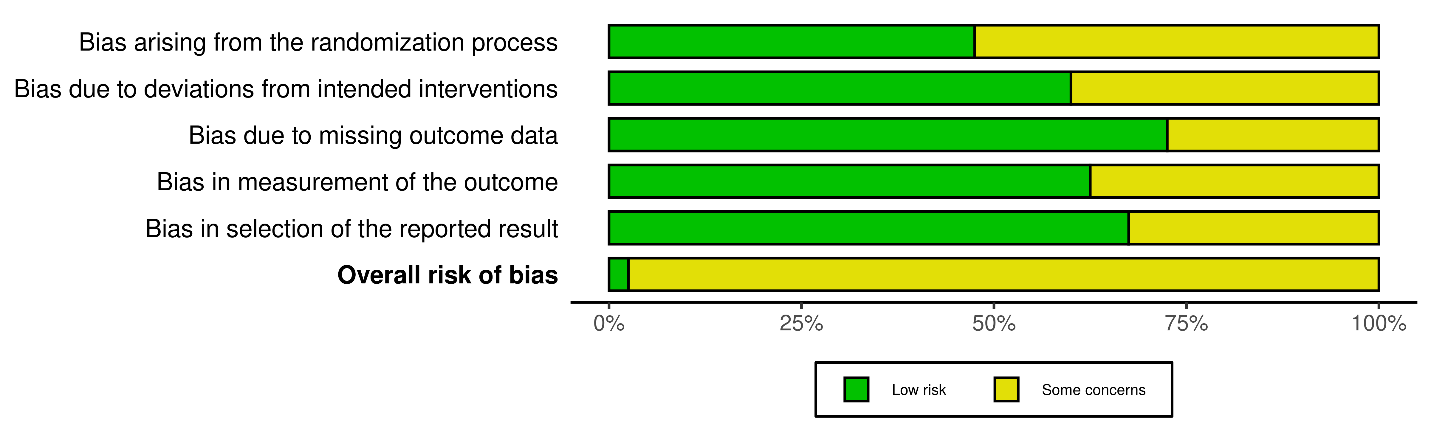
**Figure S8:** Risk of Bias Summary
