# Supplementary material for: Inflammatory biomarker response to GLP-1 receptor agonists versus other glucose-lowering medications in patients with type 2 diabetes: a systematic review and meta-analysis
Source: Front Endocrinol (Lausanne). 2026 Jan 15;16:1734549. doi: 10.3389/fendo.2025.1734549 (PMC12852008; doi:10.3389/fendo.2025.1734549)
Supplement: Supplementary file 2 [file DataSheet2.docx]

**Sensitivity analysis results**

**Assessment of Heterogeneity and Outliers**

The initial I² values exceeded 80% for a number of outcomes, including TNF-α (all comparisons), IL-6 (both comparisons), CRP (vs. OADs), and MDA (vs. insulin), indicating significant to substantial heterogeneity that cannot be solely attributed to sampling error. Certain studies were consistently identified as possible outliers by visual inspection of forest plots and statistical tests for subgroup differences because of:

1. Extreme standardized mean differences (e.g., Said 2018 across multiple biomarkers)

2. Markedly different measurement scales or baseline values (e.g., Wang Q 2020 for TNF-α),

3. Implausibly small variances given the biomarker (e.g., Ying 2023 for IL-6)

4. Effect directions that contrasts with the general trend (e.g., Kang 2021 for TNF-α vs. insulin, Forst 2012 for CRP).

The core pooled estimates' dependability and interpretability are compromised by the existence of these outliers and the ensuing substantial heterogeneity. To investigate the impact of these studies on the overall outcomes, specified sensitivity analyses were conducted.

**Table 1: Summary of Sensitivity Analyses on Heterogeneity and Pooled Effects**

| **Outcome & Comparison** | **Excluded Study (Reason)** | **I² Before** | **I² After** | **Pooled SMD After (95% CI)** | **Interpretation** |
| --- | --- | --- | --- | --- | --- |
| **CRP: GLP-1 vs. Insulin (Figure S.4.1)** | Gurkan 2014 (opposite direction); Kang 2021 (extreme negative effect) | 83.3% | 0.0% | -0.56 (-0.83 to -0.29) | Significant, homogeneous benefit for GLP-1. Initial heterogeneity resolved. |
| **TNF-α: GLP-1 vs. OAD (Figure S.4.2)** | Said 2018 (extreme SMD, different scale); Wang Q 2020 (different scale, opposite effect) | 93.3% | 15.8% | -0.23 (-0.66 to 0.19) | Effect becomes modest and non-significant; heterogeneity resolved. |
| **TNF-α: GLP-1 vs. Insulin (Figure S.4.3)** | Kang 2021 (extreme positive outlier, opposite direction) | 96.5% | 0.0% | -0.24 (-0.48 to 0.01) | Effect becomes modest and non-significant; heterogeneity eliminated. |
| **TNF-α: GLP-1+OAD vs. OAD (Figure S.4.4)** | Said 2018 (extreme negative outlier) | 87.1% | 40.7% | -1.08 (-1.41 to -0.74) | Significant benefit remains; heterogeneity reduced to moderate. |
| **IL-6: GLP-1+OAD vs. OAD (Figure S.4.5)** | Said 2018 (extreme effect, different measurement scale) | 90.5% | 15.3% | -0.79 (-1.26 to -0.32) | Significant benefit remains; heterogeneity largely resolved. |
| **IL-6: GLP-1 vs. OAD (Figure S.4.6)** | Ying 2023 & Said 2018 (both extreme effects, implausibly small variance) | 89.1% | 54.0% | -0.16 (-0.68 to 0.37) | Effect becomes non-significant; moderate heterogeneity remains due to subgroup differences. |
| **MDA: GLP-1 vs. Insulin (Figure S.4.7)** | Bunck 2010 (exenatide – extreme negative outlier, SMD -3.66) | 95.2% | 16.2% | -0.73 (-1.07 to -0.39) | Significant benefit remains with low heterogeneity. |

SMD: Standardized Mean Difference; CI: Confidence Interval; RA: Receptor Agonist; OAD: Oral Antidiabetic Drug.


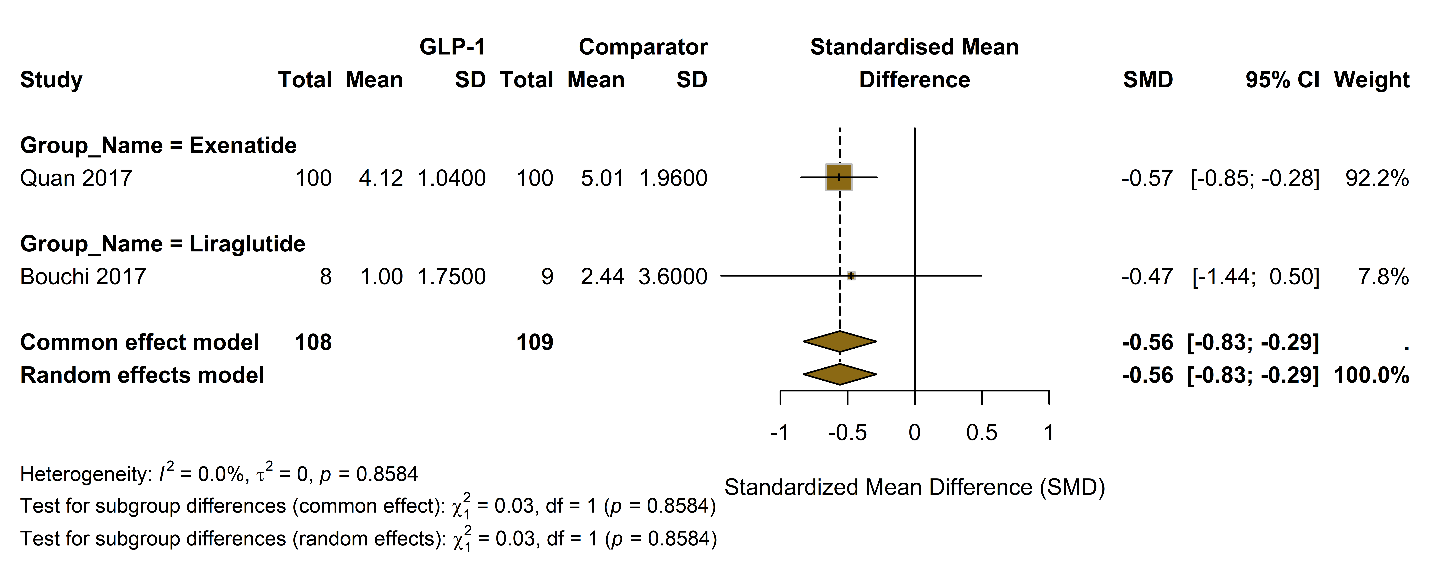


**Figure S.4.1: Forest plot of SMD in CRP levels for GLP-1 Receptor Agonists versus Insulin after sensitivity analysis**

**
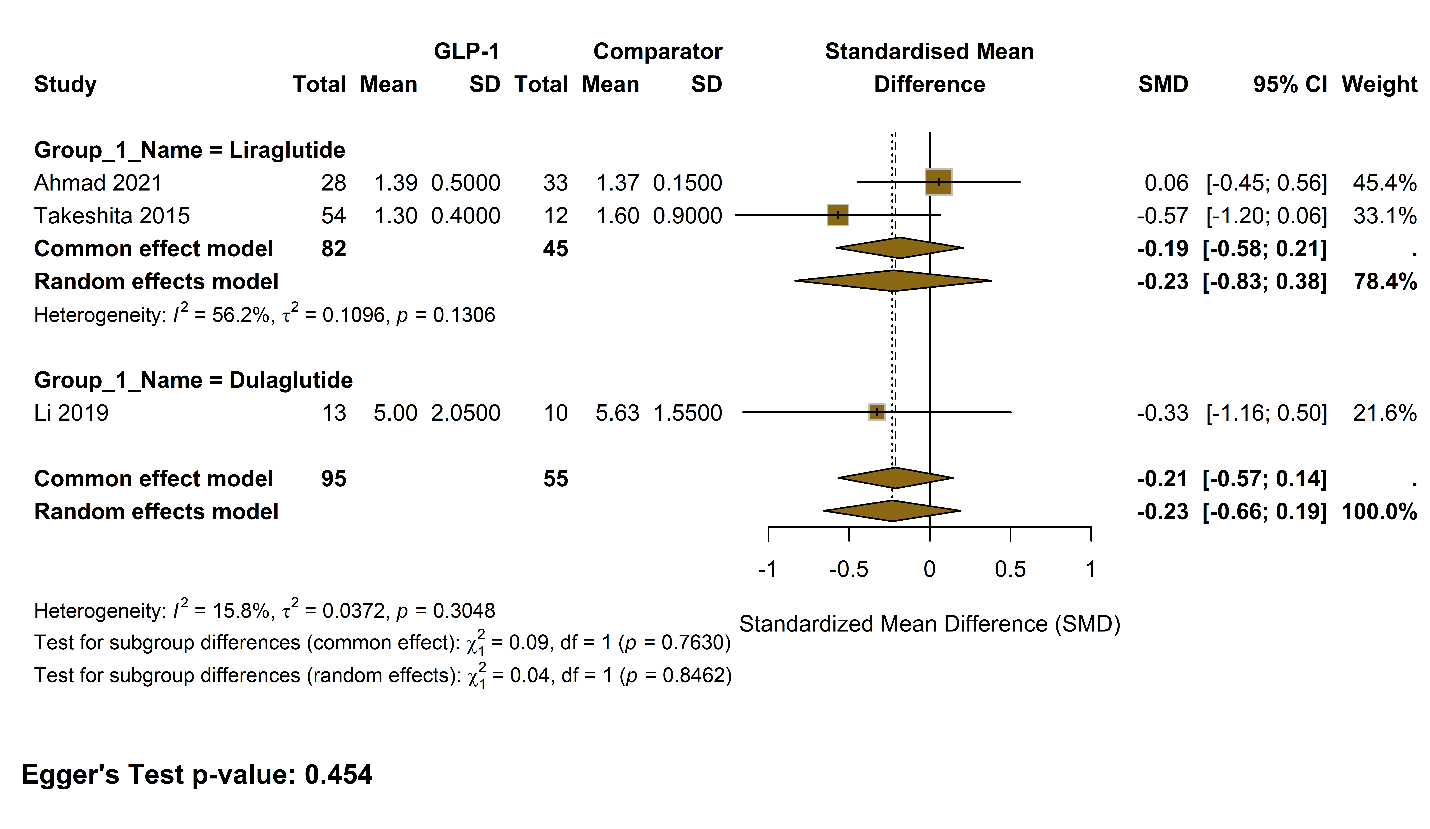
**

**Figure S.4.2: Forest plot of SMD in TNF-α levels for GLP-1 Receptor Agonists versus other Oral Antidiabetic Drugs after sensitivity analysis**

**
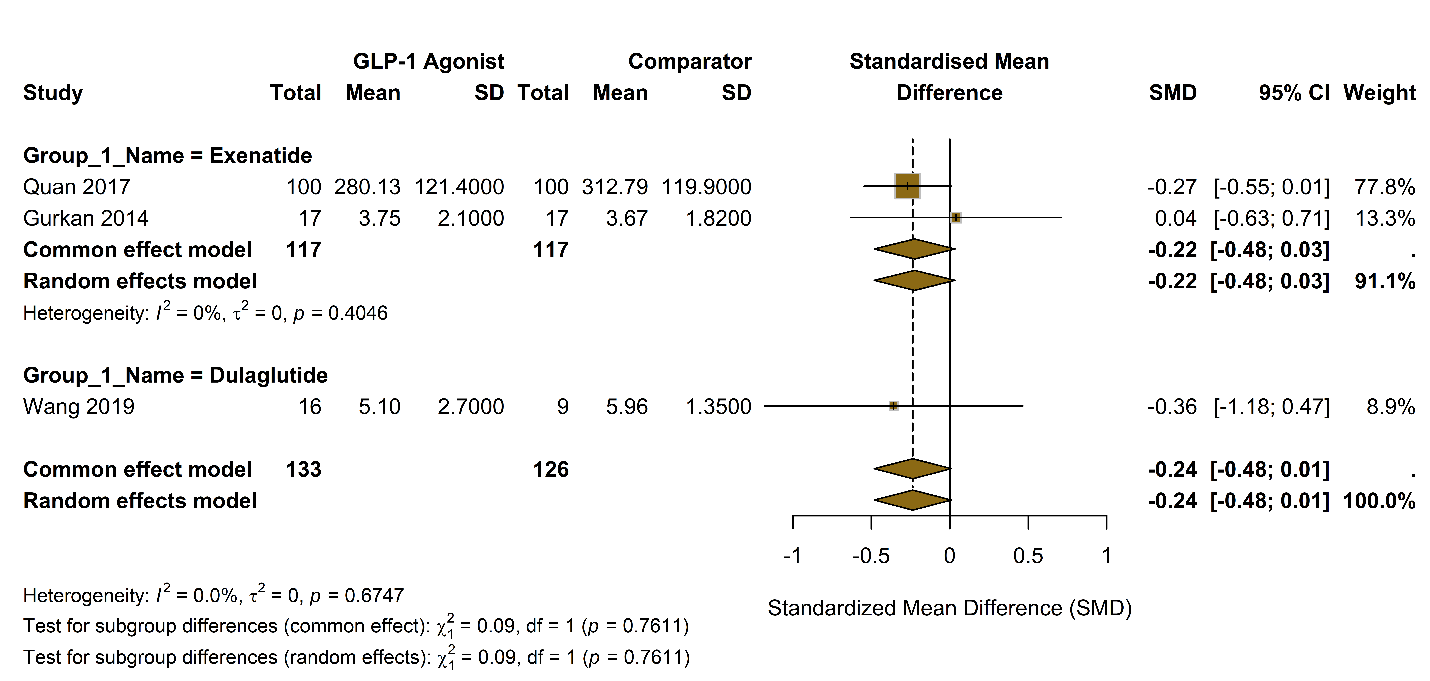
**

**Figure S.4.3: Forest plot of SMD in TNF-α levels for GLP-1 Receptor Agonists versus insulin after sensitivity analysis**


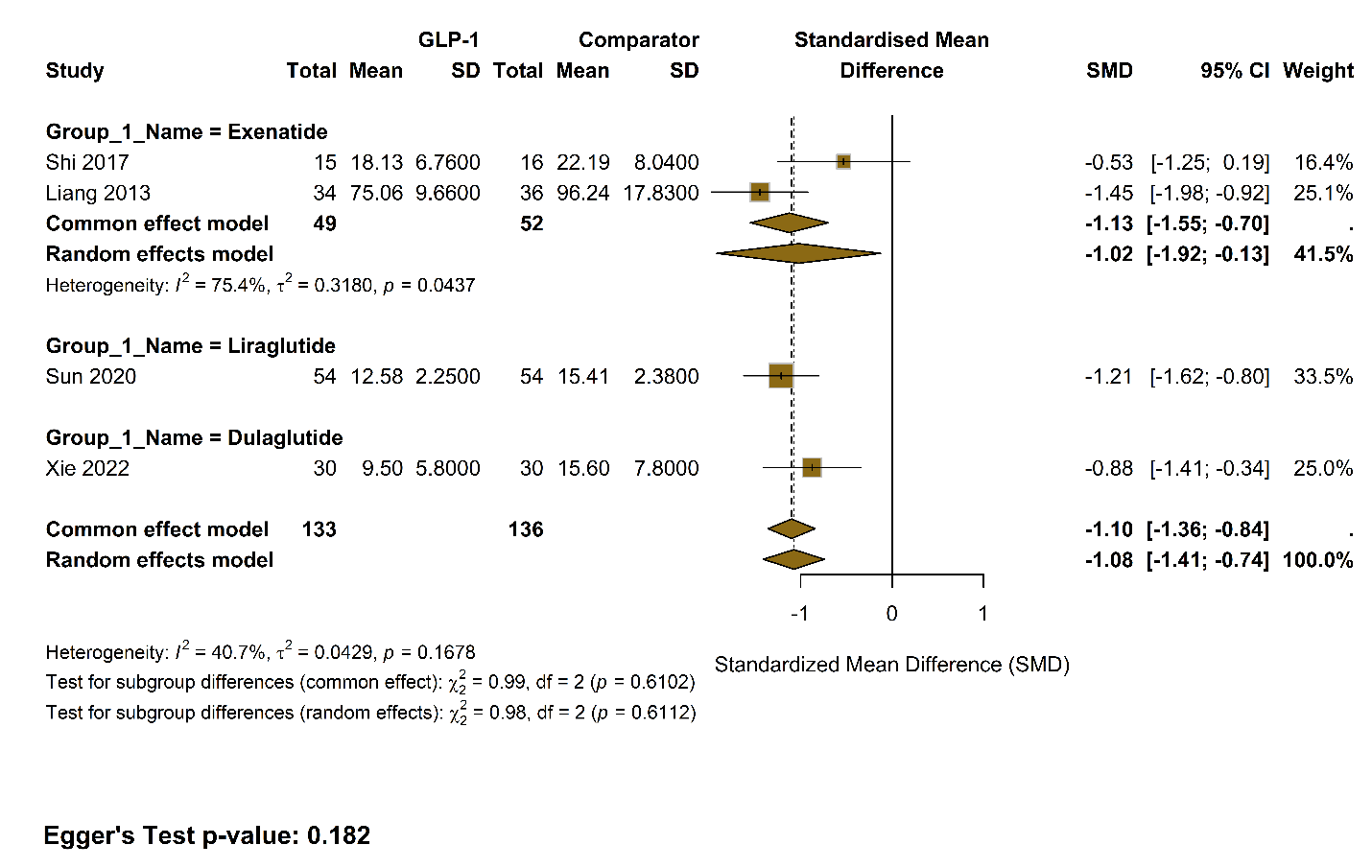


**Figure S.4.4: Forest plot of SMD in TNF-α levels for GLP-1 Receptor Agonists plus OAD versus OAD after sensitivity analysis**

**
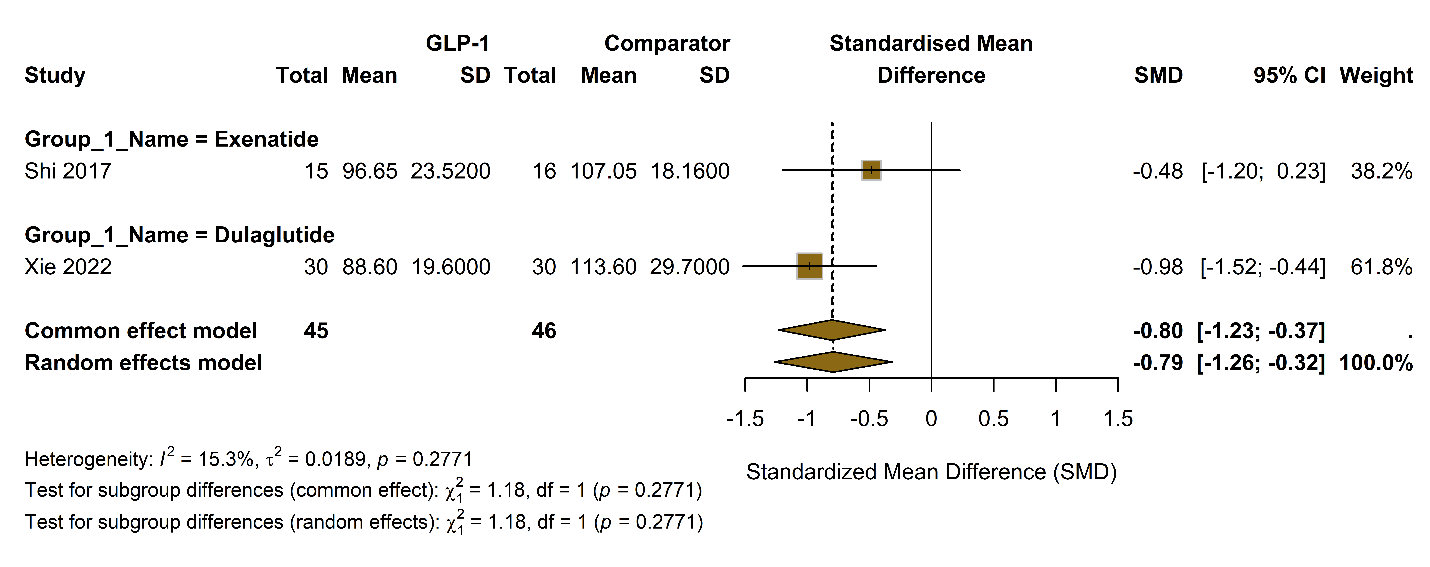
**

**Figure S.4.5: Forest plot of SMD in IL-6 levels for GLP-1 Receptor Agonists plus OAD versus OAD after sensitivity analysis**

**
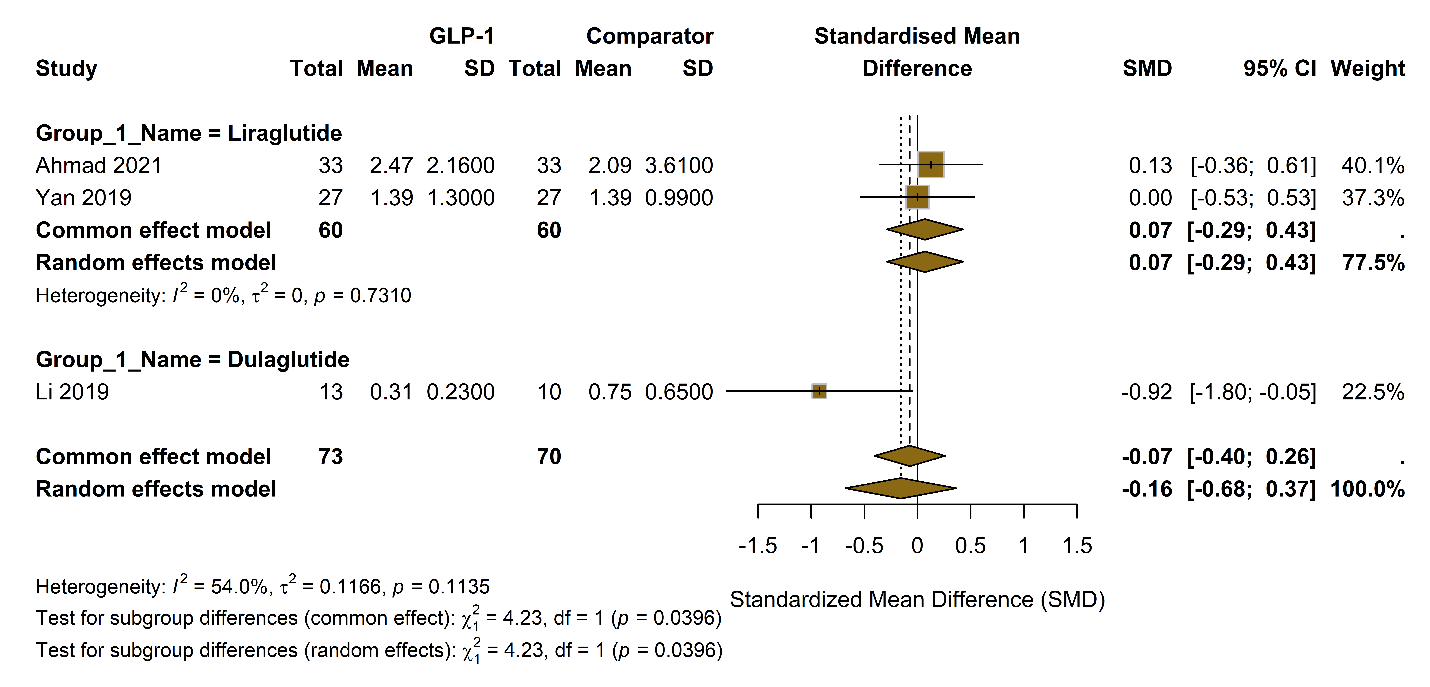
**

**Figure S.4.6: Forest plot of SMD in IL-6 levels for GLP-1 Receptor Agonists versus OAD after sensitivity analysis**

**
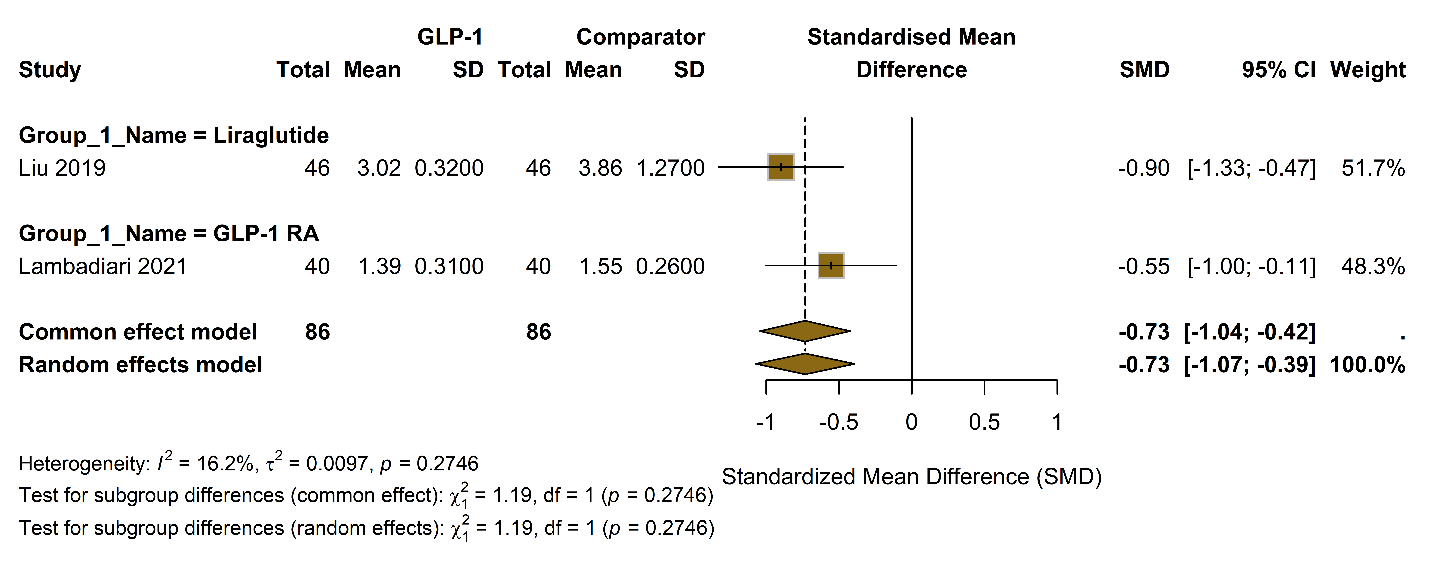
**

**Figure S.4.7: Forest plot of SMD in MDA levels for GLP-1 Receptor Agonists versus insulin after sensitivity analysis**
