# Supplementary material for: Inflammatory biomarker response to GLP-1 receptor agonists versus other glucose-lowering medications in patients with type 2 diabetes: a systematic review and meta-analysis
Source: Front Endocrinol (Lausanne). 2026 Jan 15;16:1734549. doi: 10.3389/fendo.2025.1734549 (PMC12852008; doi:10.3389/fendo.2025.1734549)
Supplement: Supplementary file 3 [file DataSheet3.docx]

**Detailed search strategy of each database**

**Search Completion Date**

Last search conducted: August 1, 2025

**Databases Searched**

PubMed/MEDLINE

Embase

Cochrane Central Register of Controlled Trials (CENTRAL)

Web of Science

**Search Filters and Restrictions**

Language: English only

Study Type: Humans only

Date Range: Inception to August 1, 2025

Publication Type: Randomized controlled trials (RCTs)

**PubMed/Medline**

| 1 | GLP 1RA*[Title/Abstract] OR  GLP 1 RA*[Title/Abstract] | 4050 |
| --- | --- | --- |
| 2 | (Glucagon-Like Peptide 1 [MeSH Terms] OR Glucagon-Like Peptide 1[Title/Abstract] OR GLP-1[Title/Abstract] ) and (receptor agonist [Title/Abstract] OR analog*[Title/Abstract]) | 8501 |
| 3 | (Dulaglutide or Exenatide extended release OR Semaglutide or Ozempic OR Liraglutide OR "albiglutide" OR "lixisenatide" OR "taspoglutide) [Title/Abstract] | 12201 |
| 4 | 1 OR 2 OR 3 | 22960 |
| 5 | (reactive protein/)[Title/Abstract] | 106864 |
| 6 | (interleukin 6/)[Title/Abstract] | 75690 |
| 7 | (tumor necrosis factor alpha/)[Title/Abstract] | 104000 |
| 8 | (inflammation" OR "inflammatory") | 221320 |
| 9 | 5 OR 6 OR 7 OR 8 | 485821 |
| 10 | 4 AND 9 | 963 |
| 11 | limit 13 to humans | 602 |
| 12 | (randomized controlled trial [Title/Abstract]) OR (controlled clinical trial [Title/Abstract]) | 221523 |
| 13 | 11 AND 12 | 376 |

**Total PubMed/MEDLINE Results: 376**

**Embase**

| 1 | (GLP 1RA* or GLP 1 RA*).mp. | 20362 |
| --- | --- | --- |
| 2 | ((Glucagon-Like Peptide 1 or GLP-1 or GLP 1 or Glucagon-Like Peptide-1) and (receptor agonist or analog*)).mp. | 37930 |
| 3 | (Dulaglutide or Exenatide extended release OR Semaglutide or Ozempic OR Liraglutide OR "albiglutide" OR "lixisenatide" OR "taspoglutide).mp. | 30060 |
| 4 | 1 or 2 or 3 | 87863 |
| 5 | (C-Reactive Protein or C Reactive Protein or hsCRP or High Sensitivity C-Reactive Protein or High Sensitivity C Reactive Protein or hs-CRP).mp. | 218582 |
| 6 | (interleukin 6 or Interleukin 6).mp | 234125 |
| 7 | (Tumor Necrosis Factor-alpha or Tumor Necrosis Factor alpha or Tumor Necrosis Factor or TNF-alpha).mp. | 263417 |
| 8 | 5 or 6 or 7 | 815013 |
| 9 | 4 and 8 | 1558 |
| 10 | limit 14 to human | 1293 |
| 11 | (randomized controlled trial or controlled clinical trial or randomized or placebo or randomly or trial or clinical trials as topic).mp. | 4422243 |
| 12 | 10 and 11 | 265 |

**Total Embase Results: 265**

**CENTRAL**

| 1 | MeSH descriptor: [Glucagon-Like Peptide 1] explode all trees | 5338 |
| --- | --- | --- |
| 2 | (Glucagon-Like Peptide 1 or Glucagon Like Peptide 1 or GLP-1 or GLP 1 or Glucagon-Like Peptide-1):ti,ab,kw (Word variations have been searched) | 9463 |
| 3 | #1 or #2 | 11382 |
| 4 | (receptor agonist or analog*):ti,ab,kw (Word variations have been searched) | 204550 |
| 5 | #3 and #4 | 11634 |
| 6 | (Dulaglutide or Exenatide extended release OR Semaglutide or Ozempic OR Liraglutide OR "albiglutide" OR "lixisenatide" OR "taspoglutide). | 10548 |
| 7 | { #5 OR#6} | 20803 |
| 9 | (C-Reactive Protein or C Reactive Protein or hsCRP or High Sensitivity C-Reactive Protein or High Sensitivity C Reactive Protein or hs-CRP):ti,ab,kw | 30561 |
| 10 | (interleukin 6 or Interleukin 6 or IL6) | 28396 |
| 11 | (Tumor Necrosis Factor-alpha or Tumor Necrosis Factor alpha) | 16133 |
| 12 | #9 or #10 OR #11 | 31621 |
| 13 | #7 and #12 | 303 |

Total CENTRAL Results: 303

**Web of science**

| 1 | (((TS=(Glucagon-Like Peptide 1 or Glucagon Like Peptide 1 or GLP-1 or GLP 1 or Glucagon-Like Peptide-1)) AND TS=(receptor agonist or analog*)) OR TS=(Dulaglutide or Exenatide extended release or Semaglutide or Ozempic or Liraglutide or Lixisenatide and Preprint Citation Index (Exclude – Database) | 42615 |
| --- | --- | --- |
| 2 | (((((TS=(C-Reactive Protein or C Reactive Protein or hsCRP or High Sensitivity C-Reactive Protein or High Sensitivity C Reactive Protein or hs-CRP)) OR TS=(interleukin 6 or Interleukin 6 or IL6 OR TS=(Tumor Necrosis Factor-alpha or Tumor Necrosis Factor alpha) and Preprint Citation Index (Exclude – Database) | 865149 |
| 3 | #1 AND #2 and Preprint Citation Index (Exclude – Database) | 4055 |
| 4 | TS= (randomized controlled trial or controlled clinical trial or randomized or placebo or randomly or trial or clinical trials as topic) and Preprint Citation Index (Exclude – Database) | 982642 |
| 5 | #3 AND #4 and Preprint Citation Index (Exclude – Database) | 204 |

**Total Web of Science Results: 204**
